# Supplementary material for: Exploring charge density waves in two-dimensional NbSe2 with machine learning
Source: NPJ Comput Mater. 2026 Apr 24;12(1):239. doi: 10.1038/s41524-026-02063-4 (PMC13368586; doi:10.1038/s41524-026-02063-4)
Supplement: Supplementary file 1 — Supplementary Information [file 41524_2026_2063_MOESM1_ESM.pdf]

Supplementary Information for "Exploring Charge Density Waves in Two-Dimensional $NbSe_2$ with Machine Learning"

Norma Rivano,[1,*] Francesco Libbi,[1] Chuin Wei Tan,[1] Christopher T. S. Cheung,[2] Jose L. Lado,[3] Arash A. Mostofi,[2] Philip Kim,[4] Johannes Lischner,[2] Adolfo O. Fumega,[2,3,†] Boris Kozinsky,[1,‡] and Zachary A. H. Goodwin[1,2,5,§]

[1] *John A. Paulson School of Engineering and Applied Sciences, Harvard University, Cambridge, MA 02138, USA*
[2] *Departments of Physics and Materials and the Thomas Young Center for Theory and Simulation of Materials, Imperial College London, London SW7 2AZ, United Kingdom*
[3] *Department of Applied Physics, Aalto University, 02150 Espoo, Finland*
[4] *Department of Physics, Harvard University, Cambridge, Massachusetts 02138, USA*
[5] *Department of Materials, University of Oxford, Parks Road, Oxford OX1 3PH, United Kingdom*

CONTENTS

I. Methods 2
A. DFT settings 2
B. Training and validation dataset generation 2
1. Monolayer - large smearing 3
2. Monolayer - small smearing 3
3. Bilayer 3
C. Machine learning potential: training and validation 3
D. Phonon calculations 5

II. Monolayer $NbSe_2$ without CDWs (large smearing) 6
A. Hyperparameter Scan 6
B. Test 8

III. Monolayer $NbSe_2$, with CDWs (small smearing) 12
A. Hyperparameter Scan 12
B. Test 14

IV. Additional Results for Monolayer $NbSe_2$, with CDWs 20
A. Coexistence simulations for the hexagonal phase 20
B. Coexistence simulations for incommensurate supercells 21
C. Commensurate-Incommensurate Structures 22
D. Classical transition temperature estimates 22

V. Bilayer $NbSe_2$, with CDWs 31
A. Hyperparameter scan 31
B. Test 31

VI. Additional Results for Bilayers $NbSe_2$ 32
A. Coexistence simulations for the AB Bilayer 32
B. Coexistence simulations with the hexagonal phase for the natural stacking 33
C. Stacking of CDWs 34
D. Classical transition temperature estimates 36

VII. Refined MLIP: phonon dispersions and SSCHA critical temperature estimates 39
A. Monolayer 39
B. Bilayer 40

References 43

* Corresponding author: nrivano@g.harvard.edu
† Corresponding author: adolfo.oterofumega@aalto.fi
‡ Corresponding author: bkoz@g.harvard.edu
§ Corresponding author: zac.goodwin@materials.ox.ac.uk

I. METHODS

This section provides a detailed expansion of the computational and machine-learning methods summarized in the main text. We combine first-principles density functional theory (DFT) [1–3] calculations with machine-learning interatomic potentials (MLIPs) to model structural and vibrational properties of $NbSe_2$. Bayesian active learning (BAL) and DFT relaxations are used to generate diverse datasets for training the Allegro MLIP [4]. The trained model is interfaced with LAMMPS [5, 6] for molecular dynamics (MD) simulations, which we use for iterative training and to predict charge-density-wave (CDW) properties such as coexistence of different phases, their evolution, binding energy curves, and incommensurate structures. Phonon properties are computed by finite differences as implemented in Phonopy [7], coupled with the trained MLIP, and finite-temperature phonon properties are obtained using the Stochastic Self-Consistent Harmonic Approximation (SSCHA) [8, 9]. Details of these calculations are given below.

A. DFT settings

First-principles calculations of structures, electronic properties, and phonons were performed using the Quantum Espresso (QE) package [10, 11]. We employed Ultrasoft Pseudopotentials [12], generated using the Vanderbilt code, with a scalar-relativistic approximation for the Nb and Se atoms. The exchange-correlation functional used was vdW-DF2-c09 [13], which includes van der Waals interactions. The plane-wave basis set was truncated at a kinetic energy cutoff of 70 Ry for wavefunctions and 560 Ry for charge density. For the k-point sampling, a 27×27×1 grid was used for the primitive cell containing three atoms, and this was appropriately rescaled for larger supercells. We treated all materials as non-magnetic, assuming no spin polarization in the calculations. Electronic state occupations were treated using the Methfessel-Paxton smearing method [14], applying a Gaussian broadening of 0.005 Ry for the small-smearing case (when CDW phases are stable, and lower in energy than the pristine case) and 0.05 Ry for the large-smearing case, i.e., when CDW phases do not exist. All calculations, except for monolayer frames obtained via relaxation from random displacements, were carried out using 2D-DFT [15], as implemented in QE, which includes a Coulomb cutoff to implement the correct boundary conditions for 2D systems. For both the monolayer and bilayer systems, the simulation size along the vacuum direction was maintained at 40 Å to ensure sufficient vacuum spacing and convergence with the aforementioned cutoff.

B. Training and validation dataset generation

Our approach combines multiple methods to construct a diverse dataset for training and validation. We combined BAL with the Fast Learning of Atomistic Rare Events (FLARE)[16] scheme, alongside relaxations from random displacements. Below, we summarize the general methodology, followed by more detailed descriptions of the specific calculations, training procedures, and validation processes performed for each case.

FLARE is based on sparse Gaussian process regression to map the atomic cluster expansion (ACE) descriptors [17] to the energy, force and stress labels. Specifically, the atomic descriptor consists of two- and three-body ACE descriptors. The key hyperparameters of the FLARE BAL potential include the radial order $n_{\max} = 8$, angular order $\ell_{\max} = 5$, and cutoff radius $r_{\max} = 6$Å. The FLARE potential is built on-the-fly over the course of an active learning molecular dynamics (BALMD) run. At every step of this BALMD run, FLARE assesses the uncertainty of the new frame alongside predictions for energy, forces and stresses necessary to propagate the dynamics of the system. If the uncertainty of the frame exceeds a specified threshold, DFT is performed (using coarser, cheaper settings) on the frame to generate training data used to update the FLARE model. Otherwise, the FLARE model's predictions are used to take the MD step. The frames generated over the BAL MD run are expected to be less correlated and contribute more novel data for model training. Subsequently, more stringent DFT settings are employed to recalculate these frames (one every other) and their corresponding labels, which are then included in the final dataset.

Concerning relaxations from random displacements, this method is more intuitive. We begin with a meaningful structure and perturb the atoms from their equilibrium positions according to a Gaussian distribution with a standard deviation σ. After displacing the atoms, the system is relaxed at each step, and the resulting structure is recorded as a frame. To reduce redundancy, every third frame is selected to lower correlations between data points.

These two complementary methods allow for the exploration of a broader phase space, minimizing correlations while increasing the variety of the dataset.

Throughout this work, several training and validation datasets are employed to address distinct modeling objectives, ranging from structural reconstruction to vibrational properties and transition temperatures. To reduce the learning overhead associated with this dataset complexity and to provide a clear overview of how each dataset is constructed and used, we summarize all training and validation datasets in Table S1 of the Supplemental Material. The table

reports, for each dataset, its scope, generation strategy, intended role, key Allegro hyperparameters for the models trained on such datasets, and where it is applied in the main text or SI.

1. *Monolayer - large smearing*

The construction of a dataset consisting solely of monolayer structures with 3×3 supercells for the large-smearing case, when there is no CDW phases, was investigated first. We choose the 3×3 structures to learn from to see what is required when CDW phases are present. In this case we rely only on BAL for data generation, running FLARE with inexpensive DFT settings as a heuristic to sample structures (reduced plane-wave cutoffs and coarser k-point sampling), followed by a refinement step in which energy and forces were recalculated using more stringent DFT parameters, for which details can be found in the previous section. In total, we calculated 265 high-quality structures for the large-smearing case.

2. *Monolayer - small smearing*

For the small-smearing case, the dataset was generated in two stages. Initially, we focused on 3×3 supercells starting from the pristine phase. Approximately half of the structures were generated using FLARE, following a procedure similar to that used for the large-smearing case. The remaining (half of the) structures were generated via DFT relaxations, starting from random displacements of the normal-phase monolayer. These random displacements were applied by introducing a Gaussian distribution with a standard deviation of σ=0.2 Å. In most cases, the first 2-3 frames from the relaxation trajectory were discarded, as they were far from equilibrium and not representative of the structures we are interested in sampling. To reduce correlations between frames, only every third frame was retained. In total, 889 high-quality frames were collected.

Later, as discussed in the main text, we refined the dataset through iterative training by including incommensurate (1×1, 2×2, 4×4, 5×5) and strained supercells ($\pm 1\%$,$\pm 2\%$ on the 3×3). The strained structures were sampled using FLARE, following the same approach as previously described. The incommensurate structures were generated starting from the initial Allegro models trained on the combined BAL-relaxation dataset. These structures were generated using the method that was used to test the models (200 K NVT simulations sampling every 1 ps), with further details being given in Section II B. This refinement resulted in an additional 535 structures, for a total of 1424 structures.

3. *Bilayer*

For the bilayer systems, only BAL was employed to generate the dataset. These BAL runs started from five distinct stacking orders of a 3×3 supercell, varying layer separations for the 0° and 180° structures. The final bilayer dataset includes both the BAL frames from the previously described monolayer dataset (471 frames) and the newly generated BAL bilayer frames, resulting in a total of 1484 structures.

C. Machine learning potential: training and validation

We use the Allegro architecture to develop our MLIPs, which leverages an equivariant neural network for both accuracy and data efficiency, and is scalable due to its strict locality. During training, the model learns the generalized potential energy of the system, which is a function of atomic coordinates and a set of parameters, including energy, forces, and stresses. The models are trained on the data generated as outlined in Section I B, using 80% of the data for training and the remaining 20% for validation. The Adam optimizer was used with a learning rate of 5×10^{-4}, with other hyperparameters set to the default values from PyTorch. The learning rate was reduced by a factor of 0.5 when the validation loss has not decreased by a factor of 1×10^{-4} of its original value within 50 epochs. Training was terminated if the validation loss does not improve for 100 epochs, the learning rate has decreased beyond 1×10^{-6}, or if 1000000 epochs has been reached (which is never the case in practice). For validation and production simulations, we used a model whose weights were the exponential moving average (EMA) of the trained model's weights, with an EMA decay factor of 0.99. While the angular order $\ell_{\max}$, number of tensor product layers n_{layer}, and cutoff radius $r_{\max}$varied across different models, the other hyperparameters were fixed for all models.

The Allegro MLIP is integrated into LAMMPS,which we used to perform MD simulations, structural relaxations, and binding energy curve calculations. For MD simulations, we employed the NVT ensemble with the Nosé-Hoover

Table S1: Schematic summary of the datasets and trained MLIPs used throughout this work. For each dataset we report its scope, how it was generated, its intended role (structural reconstruction vs. vibrational properties), the key Allegro hyperparameters, and where it is used in the manuscript/SI.

Dataset	MLIP (short name)	# frames	Contents / how generated	Key hyperparameters	Primary use (paper/SI)
**Mono–Normal** (large smearing)	**M0**	265	BAL MD at 300 K in the normal state (large electronic smearing); 3×3 supercells.	$r_{\max} = 5$ Å, $l_{\max} = 4$, $n_{\text{layers}} = 3$	Normal-state validation, baseline phonons; Fig. 1 and SI Sec. II.
**Mono–CDW (structural)** (small smearing)	**M1**	1424	Initial 3×3 CDW dataset (BAL + random-displacement relaxations) plus targeted *strained* 3×3 frames $(\pm 1\%, \pm 2\%)$ and *incommensurate/rectangular* supercells (iterative training) to improve extensibility and suppress rippling artifacts.	$r_{\max} = 5$ Å, $l_{\max} = 5$, $n_{\text{layers}} = 2$	Structural reconstruction, coexistence/interconversion MD, commensurate/incommensurate relaxations; Figs. 2–3 and SI Secs. III–IV.
**Bilayer–CDW (structural)**	**B1**	1484	Subset of monolayer 3×3 frames (471) + BAL bilayer sampling (1013) spanning multiple stackings and interlayer separations at 200 K; includes $0^\circ/180^\circ$ orientations to learn interlayer coupling and stacking energetics.	$r_{\max} = 8$ Å, $l_{\max} = 4$, $n_{\text{layers}} = 2$	Bilayer binding curves, stacking energetics, coexistence MD; Figs. 4–5 and SI Secs. V–VI.
**Mono–Vib (refined)**	**V1**	462	Pruned and rebalanced monolayer dataset emphasizing larger supercells (square 3×3–8×8 and selected $3 \times n$ rectangles) to reduce small-cell bias; trained with extended cutoff to capture longer-range IFCs relevant to the soft mode.	$r_{\max} = 10$ Å(extended), $l_{\max} = 5$, $n_{\text{layers}} = 2$	Phonon dispersions and SSCHA T_{CDW} for monolayer; Fig. 6a–b and SI Sec. VIIA.
**Bilayer–Vib (refined)**	**VB1**	1475	Refined vibrational set combining the pruned monolayer dataset (Mono–Vib) with the full bilayer training set to improve the soft-mode description under interlayer coupling; trained with extended cutoff.	$r_{\max} = 10$ Å(extended), $l_{\max} = 4$, $n_{\text{layers}} = 2$	Bilayer phonons and SSCHA T_{CDW} trends; Fig. 6c–d and SI Sec. VIIB.

thermostat, using a timestep of 0.5 fs and a damping parameter of 0.05 to maintain a constant temperature. For structural relaxations, starting either from the end of an MD simulation or from a pre-prepared structure, we used the conjugate gradient minimization protocol with no convergence tolerance for energy (i.e., the minimization will not stop based on the energy tolerance alone) and a force tolerance of 10^{-8} eV/Å.

To calculate the binding energy curves, we performed reruns on the 1×1 bilayer structures used in the DFT binding energy calculations, which were pristine structures with high symmetry stackings. To test the Allegro MLIPs, we collected structures predicted by the MLIP, and used these coordinates as inputs to DFT to check the accuracy of energy and force predictions. Generally, these structures were sampled 1 ps apart and run at 200 K (unless otherwise specified), with different supercells investigated for the monolayers.

To estimate the transition temperature from the classical simulations, we initiated structures in hollow/filled configurations for the monolayer and hollow stacked on filled for the bilayer's MM stacking. The temperature was ramped up from 1 K to the desired temperature over 10 ps, after which a brief equilibration period of 10 ps was performed, before the production run of 10 ps. This allowed us to collect structures for analysis and compute the ensemble average pair correlation function between Nb-Nb and Se-Se.

D. Phonon calculations

Phonon calculations were performed using the Phonopy code [7] with a finite-difference scheme, in combination with both DFT and the trained Allegro MLIPs. To ensure consistency and allow for direct comparison, phonon dispersions were computed using equivalent supercell sizes in DFT and MLIP calculations. Unless otherwise specified, 3×3 supercells were adopted as a reference choice, since all MLIPs were trained on this cell size. For convergence analysis and to capture long-wavelength instabilities, additional calculations were carried out on larger supercells, up to 6×6. For the hollow-CDW phase, 1×1 supercells were used, and the phonon density of states (DOS) is reported instead of full dispersions, since only the Γ point was sampled.

The "simple" acoustic sum rule (ASR) correction was applied to remove residual drift in long-wavelength acoustic modes. However, effectively eliminating the characteristic *wings* near the Γ point in 2D systems would require the advanced ASR method of Lin *et al.* [18], which is not yet implemented in Phonopy.

For the SSCHA calculations [8, 9], the dynamical matrices computed with the MLIP were used as starting points. Larger supercells were necessary to achieve converged phonon spectra; hence, all SSCHA simulations were performed on 6×6 supercells for both monolayers and bilayers. The simulations were carried out in the NVT ensemble (relaxing only atomic positions) over a temperature range of 0–70 K, allowing us to capture the softening and eventual disappearance of the unstable mode associated with the CDW transition. SSCHA sampling was performed with 2000 configurations in the NVT ensemble, providing well-converged results and ensuring a consistent basis for comparison across dimensionalities.

II. MONOLAYER NBSE$_2$ WITHOUT CDWS (LARGE SMEARING)

As described in the main text, we trained an Allegro MLIP on DFT data with a large smearing -corresponding to a high electronic temperature- which prevents the formation of CDW phases and instead stabilizes the normal phase. This allows us to test how to train $NbSe_2$ on a simplified potential energy surface. We choose to study a 3×3 supercell, which is commensurate with the size of the CDW. This choice enables us to investigate how to train a model for $NbSe_2$ using this supercell, which is the size of interest for further study in this work. The details of how the structures were generated for this model and how it was trained can be found in Sections I B and I C.

A. Hyperparameter Scan

In Tab. S2, we present the validation mean absolute errors (MAE) for a hyperparameter scan for different $r_{\max}$ values, while keeping all other hyperparameters fixed, except for the $l_{\max}$ values. The hyperparameter $r_{\max}$ determines the range of interactions included in the model and is one of the key parameters. Note that validation errors here are used as a quick proxy to identify promising hyperparameters for further exploration. Once the promising ones are identified, we further test these models.

**Model**	F (Nb) / meVÅ^{-1}	F (Se) / meVÅ^{-1}	E / meV/atom	σ / meVÅ^{-3}
**332**	43.76	31.09	0.38	0.58
**432**	7.24	5.32	**0.05**	0.14
**532**	7.84	5.81	0.06	0.12
**632**	**6.54**	**4.76**	**0.05**	**0.06**
**732**	9.31	6.48	0.07	0.09
**832**	7.88	5.68	**0.05**	0.09
**932**	9.30	6.48	0.08	0.12
**1032**	8.77	6.31	0.07	0.10
**1132**	11.23	8.06	0.08	**0.06**
**1232**	8.72	6.07	**0.05**	0.07
**342**	43.41	30.80	0.37	0.26
**442**	7.26	5.28	0.05	0.16
**542**	6.57	4.98	**0.04**	**0.07**
**642**	8.03	5.84	0.06	0.11
**742**	6.70	4.90	0.05	0.08
**842**	11.16	7.95	0.08	0.14
**942**	9.47	6.99	0.08	0.11
**1042**	9.11	6.71	0.07	0.11
**1142**	**6.39**	**4.57**	**0.04**	0.09
**1242**	9.21	6.57	0.07	0.06
**352**	42.06	30.48	0.37	0.26
**452**	**7.19**	**5.24**	**0.05**	0.15
**552**	7.29	5.55	0.06	0.15
**652**	8.75	6.23	0.06	0.14
**752**	9.96	7.10	0.07	0.12
**852**	9.70	6.86	0.07	0.10
**952**	10.4	7.73	0.08	0.12
**1052**	7.78	5.46	**0.05**	0.12
**1152**	9.66	6.79	0.06	**0.09**
**1252**	8.90	6.28	0.06	0.10

Table S2: Normal phase at large smearing, i.e., no CDW formation.Validation MAEs for force, energy and stress for the models indicated. The model notation is $r_{\max}$ l_{max} and n_{layers}, such that $r_{max} = 5$ $l_{max} = 4$ and $n_{layers} = 2$ is denoted as **542**.

As can be seen, practically independent of the value of $l_{\max}$, we find that values of $r_{\max} = 4-6$ Å and $9-11$ Å give good validation errors. The length scale of the lattice parameter used is approximately 10.5 Å, which means that $r_{\max} = 4-6$ Å covers (almost) all unique environments, and $r_{\max} = 9-11$ Å is covering replicas in the environments. We find that small values of $r_{\max} = 3$ Å, which only include nearest-neighbor Nb-Se interactions, and intermediate $r_{\max} = 7-8$ Å perform less well.

In Tab. S3 we show validation errors for varying $l_{\max}$, which describes the angular resolution of the model, for different $r_{\max}$ (only for some of the values that were found to be reason in the previous scan), while keeping all other hyperparameters constant. Clearly, $l_{\max} = 0-2$ do not perform well, but larger values typically have a low error. As the cost of the model greatly increases with $l_{\max}$, it is best to use the lowest values.

**Model**	F (Nb) / meVÅ^{-1}	F (Se) / meVÅ^{-1}	E / meV/atom	σ / meVÅ^{-3}
**502**	47.85	32.74	0.48	1.95
**512**	11.91	9.02	0.08	0.18
**522**	9.03	6.65	0.07	**0.07**
**532**	7.84	5.81	0.06	0.12
**542**	**6.57**	**4.98**	**0.04**	**0.07**
**552**	7.29	5.55	0.06	0.15
**562**	7.96	5.64	0.06	0.11
**602**	30.99	27.47	0.34	1.70
**612**	15.57	11.57	0.12	0.16
**622**	10.76	7.82	0.07	0.09
**632**	**6.54**	**4.76**	**0.05**	**0.06**
**642**	8.03	5.84	0.06	0.11
**652**	8.75	6.23	0.06	0.14
**662**	7.94	5.98	0.06	0.11
**1002**	23.70	19.38	0.04	0.08
**1012**	9.88	7.82	0.06	**0.09**
**1022**	11.88	8.87	0.08	**0.09**
**1032**	8.77	6.31	0.07	0.10
**1042**	9.11	6.71	0.07	0.11
**1052**	**7.78**	**5.46**	**0.05**	0.12
**1062**	10.86	7.77	0.07	0.15
**1102**	22.46	17.84	0.20	0.15
**1112**	9.26	7.58	0.05	0.09
**1122**	10.2	7.34	0.07	0.14
**1132**	11.23	8.06	0.08	**0.06**
**1142**	**6.39**	**4.57**	**0.04**	0.09
**1152**	9.66	6.79	0.06	0.09
**1162**	8.49	6.05	0.06	0.10

Table S3: Normal phase at large smearing, i.e., no CDW formation. Validation MAEs for force, energy and stress for the models indicated.

Finally, in Tab. S4 we show how varying the number of layers in the network, n_{layers}, which controls the complexity of interactions considered, affects the validation errors. As can be seen larger values result in lower errors, but the cost of the model increases. Therefore, values of $n_{\text{layers}} = 2-3$ are reasonable.

Overall, we found using hyperparameters of $r_{\max} = 4-6$ Å or $9-11$ Å, $l_{\max} = 3-5$ and $n_{\text{layers}} = 2-3$ to be a reasonable starting point. In the next section we test a few models with these hyperparameters.

**Model**	F (Nb) / meVÅ^{-1}	F (Se) / meVÅ^{-1}	E / meV/atom	σ / meVÅ^{-3}
**541**	9.73	7.22	0.06	0.17
**542**	6.57	4.98	**0.04**	**0.07**
**543**	**6.23**	**4.44**	**0.04**	**0.07**
**1041**	10.29	7.64	0.07	**0.10**
**1042**	9.11	6.71	0.07	0.11
**1043**	**8.29**	**6.02**	**0.06**	0.14

Table S4: Normal phase at large smearing, i.e., no CDW formation. Validation MAEs for force, energy and stress for the models indicated.

B. Test

We tested a few Allegro models with different hyperparameters suggested from the validation error hyperparameter scans. Here we further investigated the **342**, **542**, **1042** and **543** models. The former was chosen as a reference benchmark, as we do not expect it to be accurate in comparison to the other models. We test these models from running MD simulations at various temperatures to collect the structures that the models predict, with their corresponding energy and force predictions, which are then compared against the DFT ground-truth.

In Fig. S1 we show this test for the **342** model. Overall, our test errors for this model are comparable to the validation errors in the previous section, albeit with the magnitude of the force and energy errors increasing slightly with temperature.

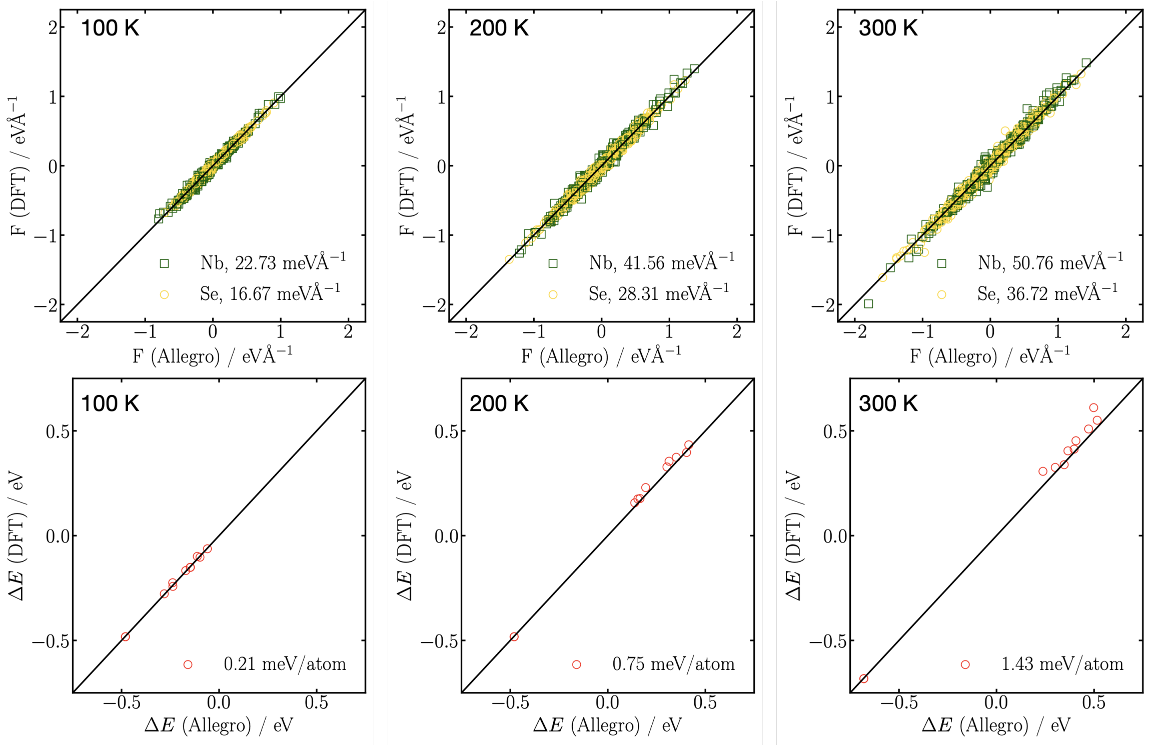

Figure S1: Normal phase at large smearing, i.e., no CDW formation. Force and energy parity plots at the indicated temperatures from the **342** model, with MAEs as indicated.

In Fig. S2 we show the test for the **542** model. At 200 K, this model's test errors are comparable to the validation errors, while the 100 K test have substantially smaller errors, owing to the smaller magnitudes of forces/energy. However, the test at 300 K is markedly worse. As the active BAL data was collected at 300 K, this is not unexpected, as structures typically need to be sampled at $\times 2$ the temperature they need to be run at, to ensure an accurate model.

In Fig. S3 we show the test for the **1042** model. Overall, the trends are similar to the **542** model, albeit with worse

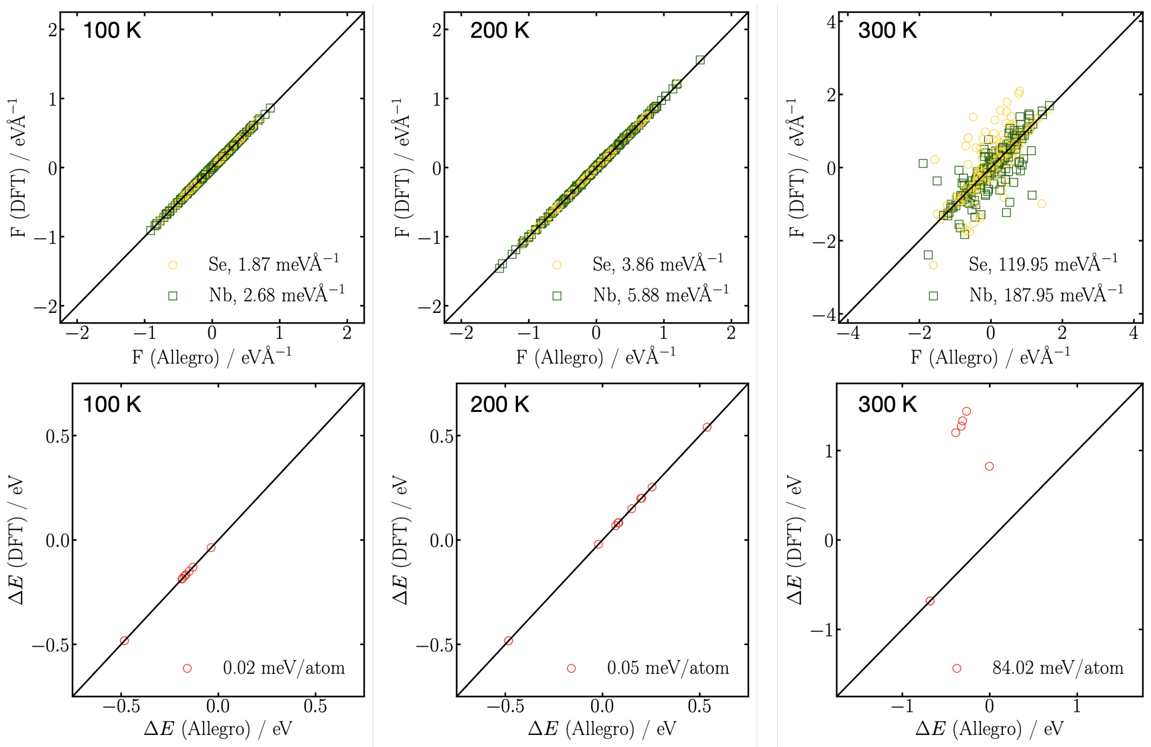

Figure S2: Normal phase at large smearing, i.e., no CDW formation. Force and energy parity plots at the indicated temperatures from the **542** model, with MAEs as indicated.

errors, especially for the 300 K runs.

Finally, in Fig. S4 we show the test for the **543** model. Again, this has similar trends to the **542** model, but with slightly better errors. Based on these results, we selected the hyperparameters from the **543** model for the final developed model.

To further validate our models, we computed phonon dispersions using Allegro+Phonopy with different hyperparameter settings and compared them with DFT+Phonopy and DFPT calculations [19, 20], as shown in Fig. S5(b) and (c), respectively. We focused on the **542**, **543**, **1042**, and **1043** Allegro models, which show excellent agreement with both DFT and DFPT results.

In Fig. S5(a), we first verify that phonon dispersions are sufficiently converged using a 3×3 supercell. To this end, we employ one of the best-performing Allegro models and compare results with those obtained using 6×6 and 9×9 supercells. The same convergence test was carried out for DFT, comparing 3×3 and 4×4 supercells. Panel (b) then compares phonon dispersions from various Allegro models to DFT, computed using finite differences with Phonopy and a 3×3 supercell. All models reproduce DFT phonons well, with no significant improvement observed upon increasing $r_{\max}$. Considering both accuracy and computational efficiency, we selected the **543** model as the optimal compromise. This comparison highlights how tuning the primary hyperparameters can either improve or slightly degrade agreement with DFT, depending on the specific model.

Finally, panel (c) presents the DFPT phonon dispersions, which, as expected, closely match the DFT results shown in panels (a) and (b). These calculations were performed using the same underlying DFT settings and a 12×12 q-grid with a phonon self-consistency threshold of 1×10^{-20} Ry^2.

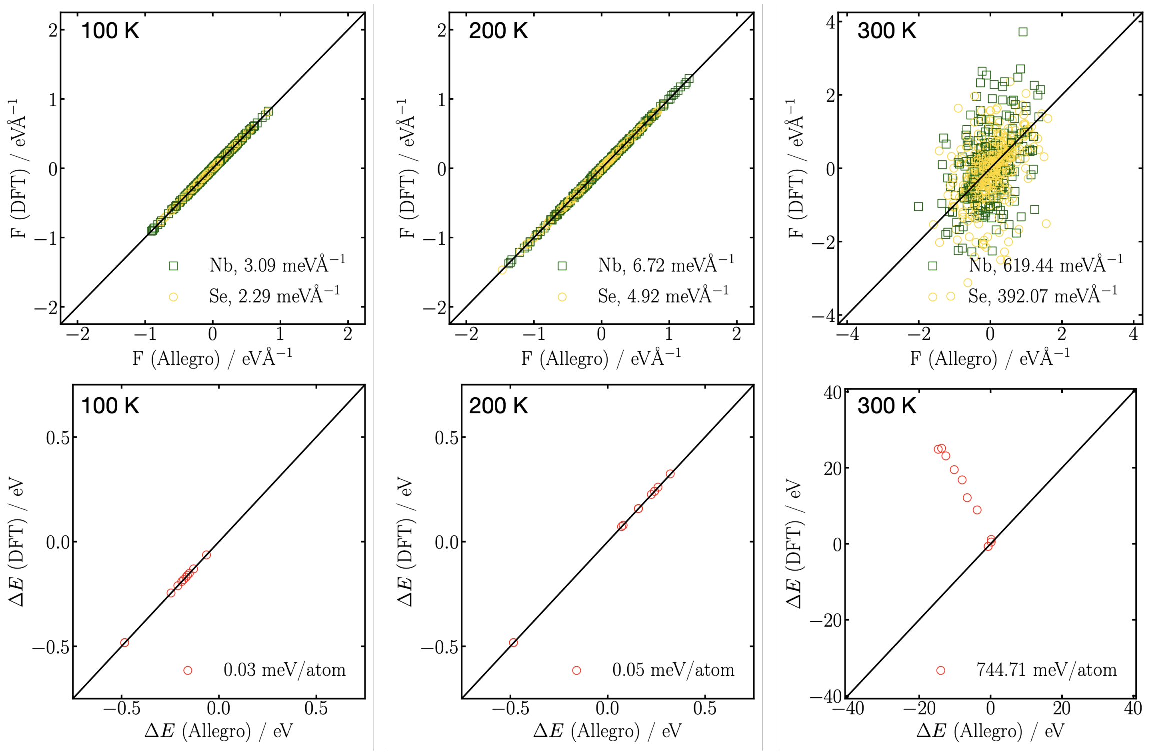

Figure S3: Normal phase at large smearing, i.e., no CDW formation. Force and energy parity plots at the indicated temperatures from the **1042** model, with MAEs as indicated.

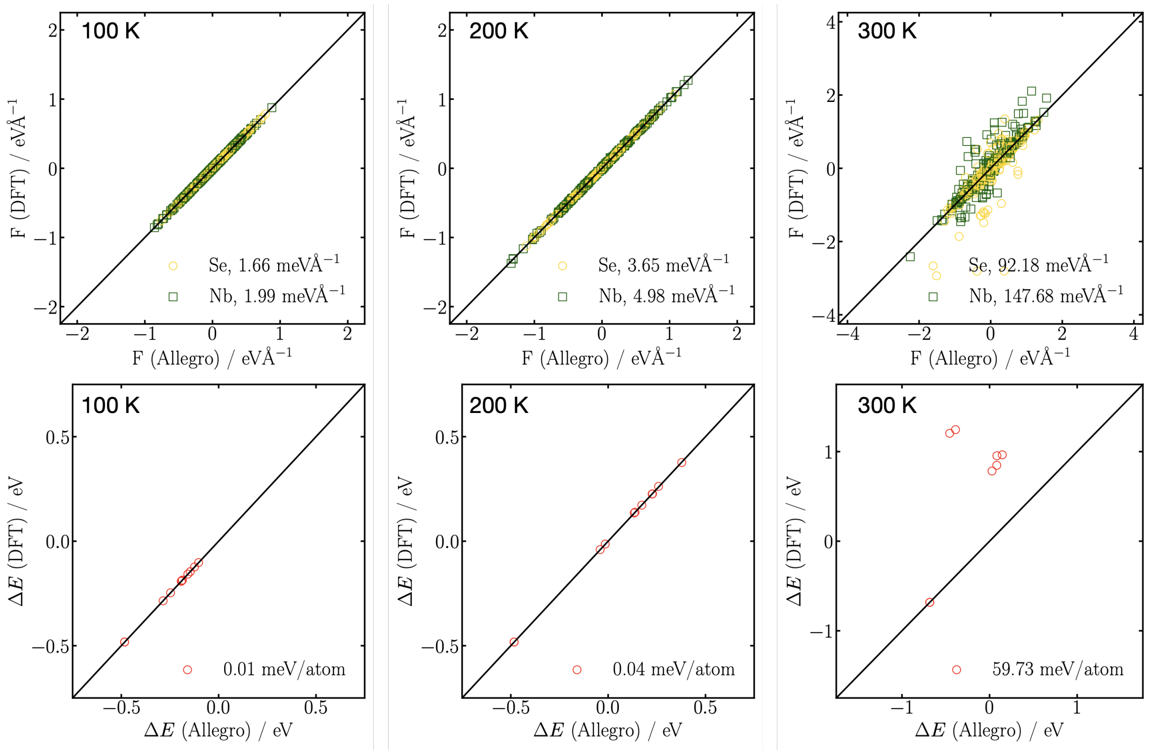

Figure S4: Normal phase at large smearing, i.e., no CDW formation. Force and energy parity plots at the indicated temperatures from the **543** model, with MAEs as indicated.

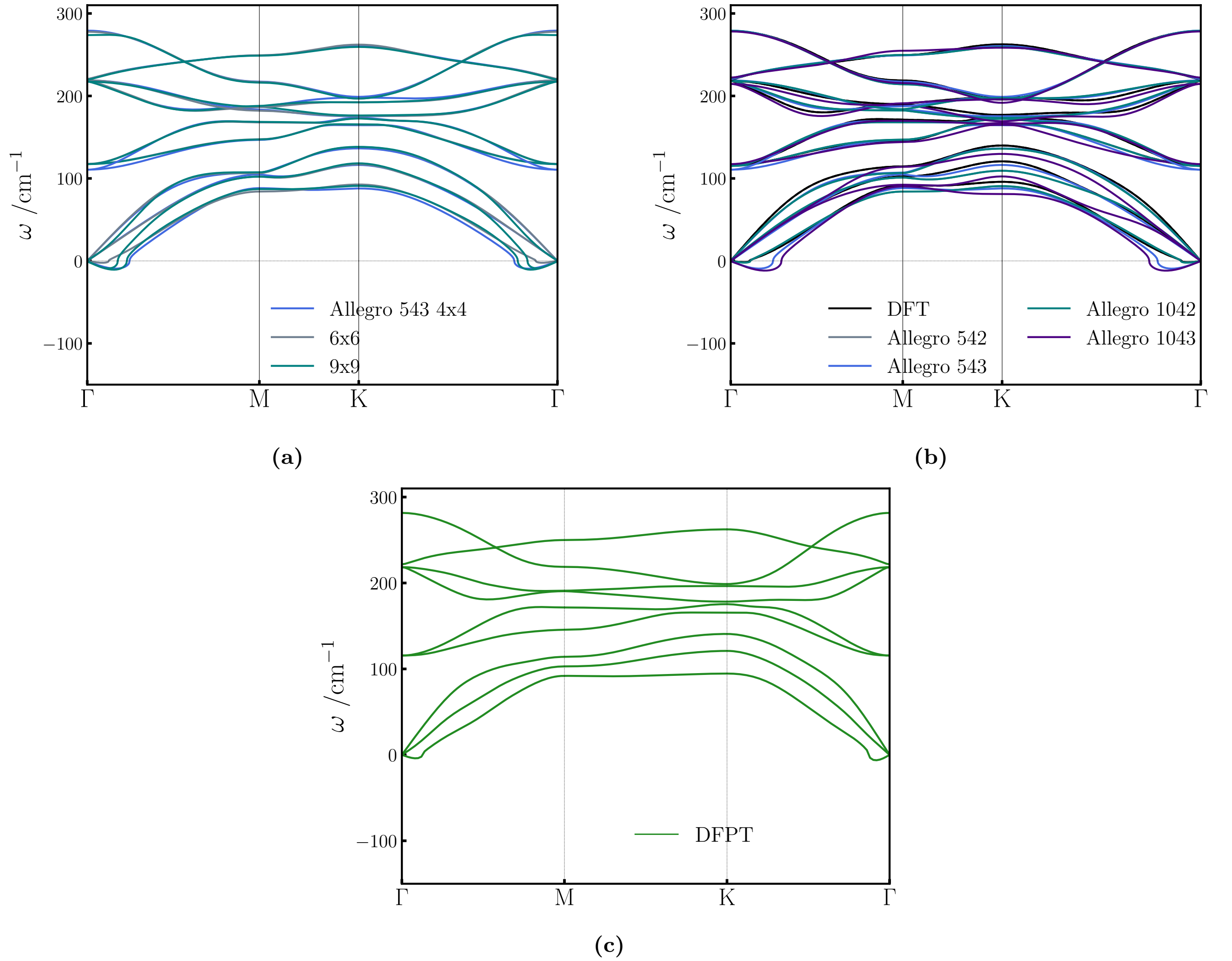

Figure S5: Comparison of phonon dispersions obtained from various models and DFT for the normal phase with large smearing (i.e., no CDWs). **a** - Phonon dispersions computed using the 543 Allegro model and finite differences with Phonopy for supercell sizes of 4×4, 6×6 and 9×9, demonstrating convergence behavior. **b** - Phonon dispersions predicted by various Allegro models (542, 543, 1042, and 1043) compared against DFT results, using finite differences with Phonopy and a 3×3 supercell. **c** - DFPT phonon dispersions, calculated using a 12×12 q-grid.

III. MONOLAYER NBSE$_2$, WITH CDWS (SMALL SMEARING)

In this section, we describe the training procedure and hyperparameter scans for monolayer $NbSe_2$ with CDW phases, i.e., with a small-smearing such that the CDW phases are preferred over the pristine structure. We begin with a hyperparameter scan on an initial dataset of 889, followed by tests and iterative training necessary for refining this model. More details of how the structures were generated for this model and how it was trained can be found in Sections I B and I C.

A. Hyperparameter Scan

In Tab. S5 we present the validation errors for training Allegro models on the dataset of 889 structures (combined dataset, i.e., BAL+relaxation from random displacements), varying $r_{\max}$ for different $l_{\max}$, while keeping all other hyperparameters fixed. We find for $l_{\max} = 3$, all values of $r_{\max}$ yield relatively low errors, except for $r_{\max} = 5$ and $r_{\max} = 7$, but with $r_{\max} = 12$ performing particularly well. For $l_{\max} = 4$, the errors are overall better than $l_{\max} = 3$, with different values of $r_{\max}$ performing well/bad. Finally, for larger $l_{\max}$, similar observations hold, again with slightly better errors. Overall, we find different combinations of $r_{\max}$-$l_{\max}$ yield low errors, indicating that multiple hyperparameter configurations can effectively minimize validation error.

Model	F (Nb) / meVÅ^{-1}	F (Se) / meVÅ^{-1}	E / meV/atom
**432**	33	20	0.28
**532**	61	38	0.62
**632**	38	24	0.32
**732**	49	33	0.51
**832**	37	24	0.31
**932**	37	24	0.31
**1032**	28	18	**0.21**
**1132**	32	20	0.26
**1232**	**27**	**16**	0.23
**442**	31	18	**0.24**
**542**	40	27	0.37
**642**	51	33	0.46
**742**	37	25	0.43
**842**	28	18	0.25
**942**	35	23	0.32
**1042**	36	22	0.30
**1142**	**26**	**15**	0.25
**1242**	29	18	0.26
**452**	31	18	0.24
**552**	35	21	0.31
**652**	35	22	0.31
**752**	47	31	0.46
**852**	28	18	0.26
**952**	41	27	0.38
**1052**	30	18	0.28
**1152**	30	19	0.25
**1252**	**25**	**14**	**0.19**

Table S5: Normal phase at small smearing, i.e., unstable to CDW formation. MAEs respectively for the forces acting on the Nb and Se atoms and the energy per atom. Varying the $r_{\max}$ for a given set of $l_{\max}$, as indicated.

In Tab. S6, we present the validation errors for models trained with varying $l_{\max}$ for different $r_{\max}$ values, while keeping all other hyperparameters fixed. As shown, small $l_{\max}$ values of $0 - 2$ generally yield higher validation errors. For small $r_{\max}$, intermediate $l_{\max}$ values appear to be more effective. In contrast, for large $r_{\max}$, only intermediate

l_{max} settings are possible, which typically have the lowest errors.

**Model**	F (Nb) / meVÅ^{-1}	F (Se) / meVÅ^{-1}	E / meV/atom
**502**	87	53	1
**512**	54	33	0.45
**522**	51	31	0.43
**532**	61	38	0.62
**542**	40	27	0.37
**552**	35	21	0.31
**562**	**30**	**19**	0.30
**572**	**30**	20	**0.29**
**582**	33	21	0.31
**592**	32	20	0.30
**5102**	53	33	0.70
**602**	81	51	0.88
**612**	105	58	1.19
**622**	48	32	0.48
**632**	38	24	0.32
**642**	51	33	0.46
**652**	**35**	**22**	**0.31**
**662**	46	29	0.41
**702**	78	52	0.95
**712**	61	42	0.73
**722**	42	29	0.44
**732**	49	33	0.51
**742**	37	25	0.43
**752**	47	31	0.46
**762**	**35**	**24**	**0.38**
**1002**	55	38	0.50
**1012**	31	18	**0.18**
**1022**	36	24	0.29
**1032**	28	18	0.21
**1042**	36	22	0.30
**1052**	30	18	0.28
**1062**	**18**	**13**	5.10
**1102**	54	35	0.47
**1112**	33	20	0.22
**1122**	29	19	0.22
**1132**	32	20	0.26
**1142**	26	**15**	0.25
**1152**	30	19	0.25
**1162**	**27**	17	**0.20**

Table S6: Normal phase at small smearing, i.e., unstable to CDW formation. MAEs respectively for the forces acting on the Nb and Se atoms and the energy per atom. Varying the l_{max} for a given set of r_{max}, as indicated.

Finally, in Tab. S7 we present the validation errors for varying number of layers, n_{layers}, at different r_{max}. As expected, based on the previous findings, increasing the number of layers generally improves the performance of the model, but this also increases its computational cost. Based on a balance between accuracy and efficiency, $n_{layers} = 2–3$ is found to be a reasonable choice.

From these tests, it is apparent that $l_{max} = 3 - 6$ is needed, and we choose to use $n_{layers} = 2$, and then the value of r_{max} depends exactly on the value of $l_{max} = 3 - 6$ chosen. As we previously used $r_{max} = 5$, we choose this value, and $l_{max} = 5 - 6$ are reasonable for this $r_{max} = 5$. Larger values of l_{max} do have lower validation errors, but the models

Model	F (Nb) / meVÅ^{-1}	F (Se) / meVÅ^{-1}	E / meV/atom
**541**	42	**25**	0.37
**542**	40	27	0.37
**543**	**38**	26	**0.36**
**1041**	29	18	**0.20**
**1042**	36	22	0.30
**1043**	**28**	**17**	0.23

Table S7: Normal phase at small smearing, i.e., unstable to CDW formation. MAEs respectively for the forces acting on the Nb and Se atoms and the energy per atom. Varying the n_{layers} for a given set of $r_{\max}$ and $l_{\max}$, as indicated.

are more expensive to run, so we refrain from using these parameters. We also found, similar to the large smearing case, that $r_{\max} = 10$ would also yield low validation errors, but we choose to purse the $r_{\max} = 5$ model.

	Model	F (Nb) / meVÅ^{-1}	F (Se) / meVÅ^{-1}	E / meV/atom
**RRD**	**552**	34	20	0.40
	**652**	26	16	0.24
	**752**	38	27	0.59
	**852**	23	15	0.20
	**952**	23	14	0.28
	**1052**	22	14	0.18
	**1152**	21	13	0.21
	**1252**	**20**	**12**	**0.26**
**BAL**	**552**	35	21	0.31
	**652**	35	22	0.31
	**752**	47	31	0.46
	**852**	28	18	0.26
	**952**	41	27	0.38
	**1052**	30	18	0.28
	**1152**	30	19	0.25
	**1252**	**23**	**13**	**0.17**

Table S8: Normal phase at small smearing, i.e., unstable to CDW formation. MAEs respectively for the forces acting on the Nb and Se atoms and the energy per atom. Varying the $r_{\max}$ for a given set of $l_{\max}$, as indicated. The **RRD** section are models trained only on the relaxations from random displacements, and the **BAL** are models only trained on the Bayesian active learning data.

For the sake of discussion, in Tab. S8 we report similar analysis for validation errors for the model trained on BAL data only. This dataset is half the size of the combined dataset and includes less diversity since the frames collected from relaxations after random displacements are missing. Same comments are valid here with respect to the combined dataset concerning the absence of clear trends; however validation errors are reduced as a consequence of the two key differences enumerated above, confirming the point raised in the main paper, that is that for relatively small dataset diversity of the frames is key and size of course can improve, as expected, model performances.

Based on the insights collected so far, for the final model (refined by including strained structures and incommensurate supercells), we avoid performing a thoughtful scan, as we identified already an $r_{\max}$=5, $l_{\max}$=5, n_{layers}=2 as reasonable hyperparametrs.

B. Test

Firstly, we tested the **552** model by performing MD simulations at 200 K to collect structures for the 3×3 supercell, along with their respective energy and force predictions. These predictions were then compared to the ground-truth

results from DFT. In Fig. S6, we show the resulting force and energy parity plots (MAEs). As can be seen good errors were obtained, comparable to the validation errors.

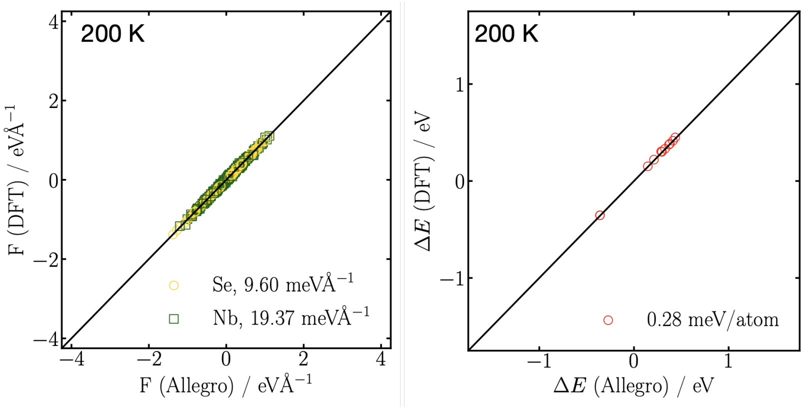

Figure S6: Normal phase at small smearing, i.e., unstable to CDW formation. Force and energy parity plots at the indicated temperatures from the **552** model on a 3×3, with MAEs as indicated.

The aim is to apply this potential to supercells of the CDWs, such as 6×6 and 9×9 or larger, as well as to supercells which are not commensurate with the CDWs, such as 1×1 and 2×2. To start, we tested how our potential would perform on these tasks. For the supercells commensurate with the CDWs, good extensibility was observed for both the normal and CDW phases, but only when these phases were prepared and relaxed close to their equilibrium configurations. When initial structures were generated with filled and hollow CDWs, and MD was run followed by relaxation, structures like the one in Fig. S7 were found with a lower energy than the normal, filled and hollow phases. This suggests that Allegro struggled with the extensibility of CDW phases, which could be a result of the chosen hyperparameters or architecture, or it could be a data issue. We note here—and this applies throughout the text—that all structural visualizations were generated using Ovito [21]. Nb–Nb bonds are shown if the atoms are within a distance of 3.45Å.

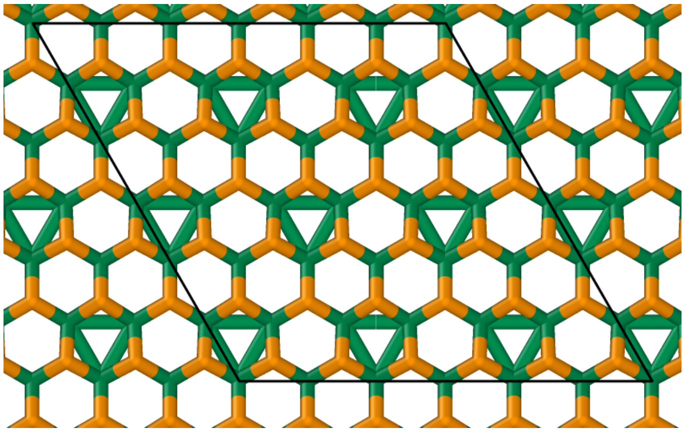

Figure S7: Structure predicted by the **552** model which was lower in energy than the hollow/filled phases.

Next we tested the model's performance on supercells that are not commensurate with the CDW phases, specifically for 1×1, 2×2, 4×4 and 5×5 structures, running MD at 200 K. In Fig. S8, we show the resulting force parity plots. As seen, the model performs well for the 1×1 structures, but the force MAEs increase for the 2×2 structures, and further degrade for the 4×4 and 5×5 supercells. Fig. S9 presents the corresponding energy parity plots. Similarly, the 1×1 structures have good energy MAEs, similar to those of the 3×3 structures used for training, whereas, 4×4 and 5×5 structures have energy MAEs larger than 1 meV/atom, and the 2×2 structures have errors exceeding 2 meV/atom. In all cases, the DFT energy is higher than the Allegro model, suggesting some systematic error in the model.

As the model accuracy requires energy MAEs smaller than 1 meV/atom to be able to distinguish between the normal and CDW phases, and since we wish to perform supercell (both commensurate and incommensurate) calculations on

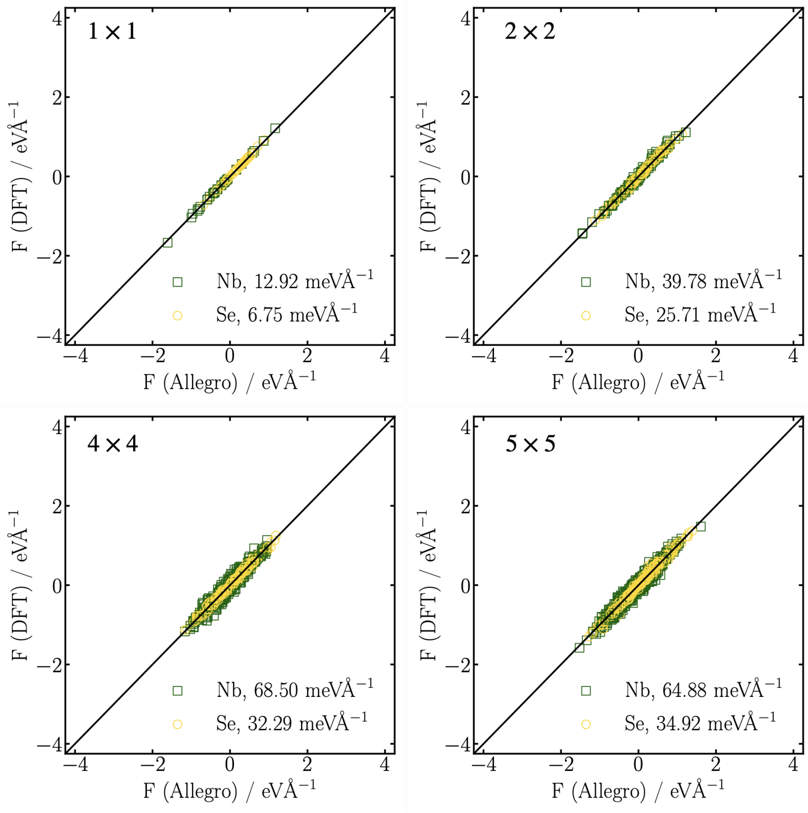

Figure S8: Force parity plots for the indicated structures from the **552** model at 200 K, with MAEs as indicated.

the CDW structures, we perform iterative training to refine our model. We keep the base-line dataset of 889 structures, but to this we add the frames from the previous test, in addition to additional relaxations from 1×1 and 2×2 supercells starting from random displacements (following the same procedure initially outlined). Therefore, we now have a new dataset, containing 1224 frames.

We tested this new model in the same way as before. For the 3×3 structure, the new model had similar test errors to before, as seen in Fig. S10, so the addition of the new structures didn't substantially reduce (or enhance) the performance. In Fig. S11 we show the corresponding parity plots for the incommensurate structures. For the 1×1 case, the errors remain good, and the 2×2 structures now show errors comparable to the 3×3 case. Force errors for the 4×4 and 5×5 structures have improved, though not significantly. This is perhaps not surprising, as more data was added for the 1×1 and 2×2 structures compared to the 4×4 and 5×5 structures. In Fig. S12 the energy errors are reported, where good values are found for 1×1, 2×2 and 4×4 structures, with energy values close to the diagonal. For the 5×5 case, an energy MAE of 1.02 meV/atom was found, which is close to the desired accuracy; however, as in previous tests, all DFT energy values are higher than the Allegro predictions.

Upon testing these models further for larger supercells (larger than 9×9), we observed significant rippling, as shown in Fig. S13, where the Allegro energy dropped to unexpectedly low values. To alleviate this issue, we performed BAL on 3×3 structures with strains of $\pm1\%$ and $\pm2\%$. In total we used 200 frames, 50 from each of these BAL runs. In addition, we further included the 4×4 and 5×5 structures generated in the previous tests, and added these to the dataset. Therefore, in total, we have 1424 structures.

Finally, we tested this refined model in a similar way to the previous iterations. In Fig. S14 we show the force and energy parity plots from the 3×3 structures at 200 K. The addition of the strained frames appears to have caused slightly worse force and energy errors, although they are still within the desired accuracy.

In Fig. S15 we show the force parity plots for the incommensurate structures generated at 200 K. The force errors found are similar to the previous iterations, which is perhaps not surprising as mainly 3×3 data was added. The corresponding energy parity plots are shown in Fig. S16, where again similar results are obtained to the previous iteration of model, albeit with slightly improved energy errors for the 5×5 structures.

Moreover, upon testing this model further on large commensurate and incommensurate structures, we found no rippling or unexpected shorter periods of periodic lattice distortions. Therefore, we believe that adding in incommensurate structures (such as 1×1-5×5) and strained monolayers is important for having extensible models, which do

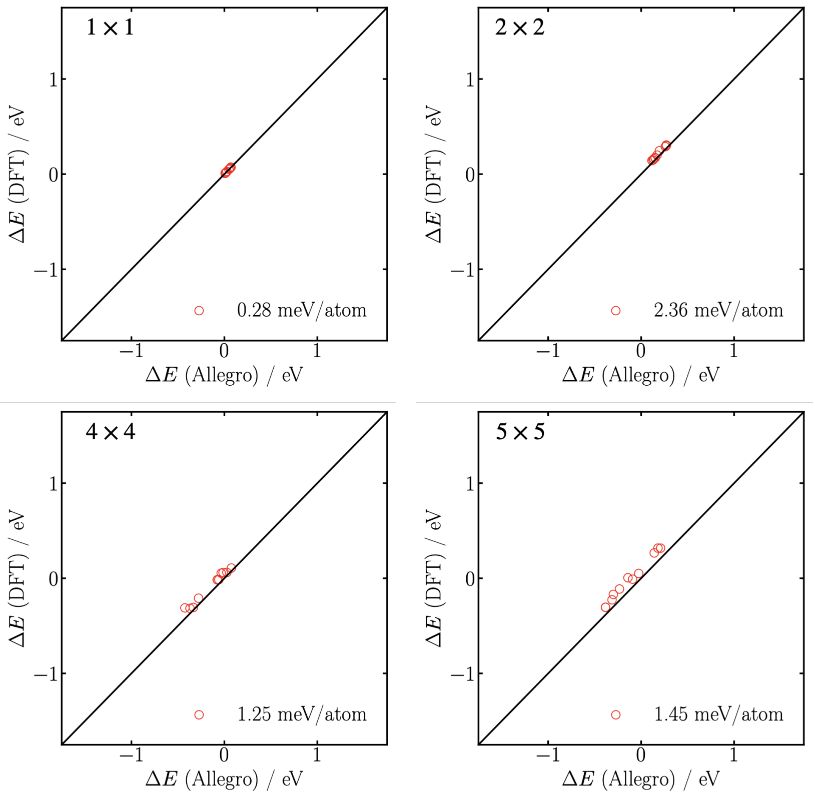

Figure S9: Energy parity plots for the indicated structures from the **552** model at 200 K, with MAEs as indicated.

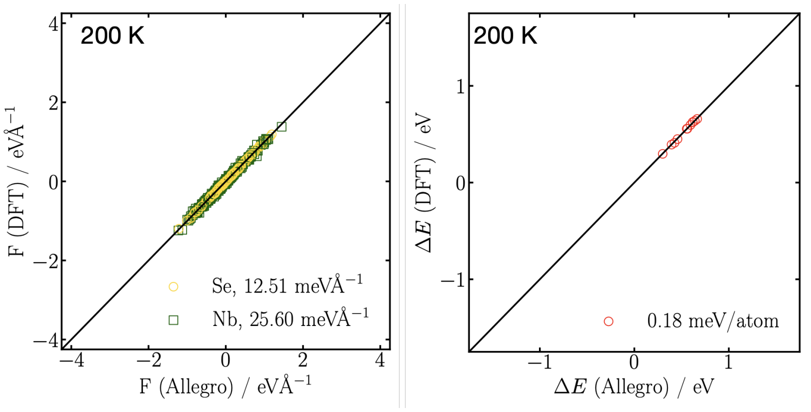

Figure S10: Force and energy parity plots at the indicated temperatures from the **552** model after including incommensurate structures on a 3×3, with MAEs as indicated.

not ripple and that can be used for incommensurate structures.

To further test this iteration of the Allegro model, we ran MD with 3×1, 3×2, 3×4 and 3×5 structures at 200 K to collect structures and compare against the DFT ground-truth. Note that these supercells are not in the training/validation set, which means this is a test of extrapolation for the developed model. In Fig. S17 we show the force parity plots for these structures and in Fig. S18 we show the energy parity plots. Clearly, the errors in these plots are similar to those of the incommensurate structures used for training, which means the model is generalizing reasonably well to unknown incommensurate structures.

As we have trained on incommensurate structures both smaller and larger than the CDW phase, we hope that any incommensurate structure larger than 6×6 is a combination of the CDW phases and the smaller incommensurate structures, such that there is no need to include all possible incommensurate structures to train a reliable model.

These tests were all performed for the **552** models. We also identified the **1042** to be another good candidate.

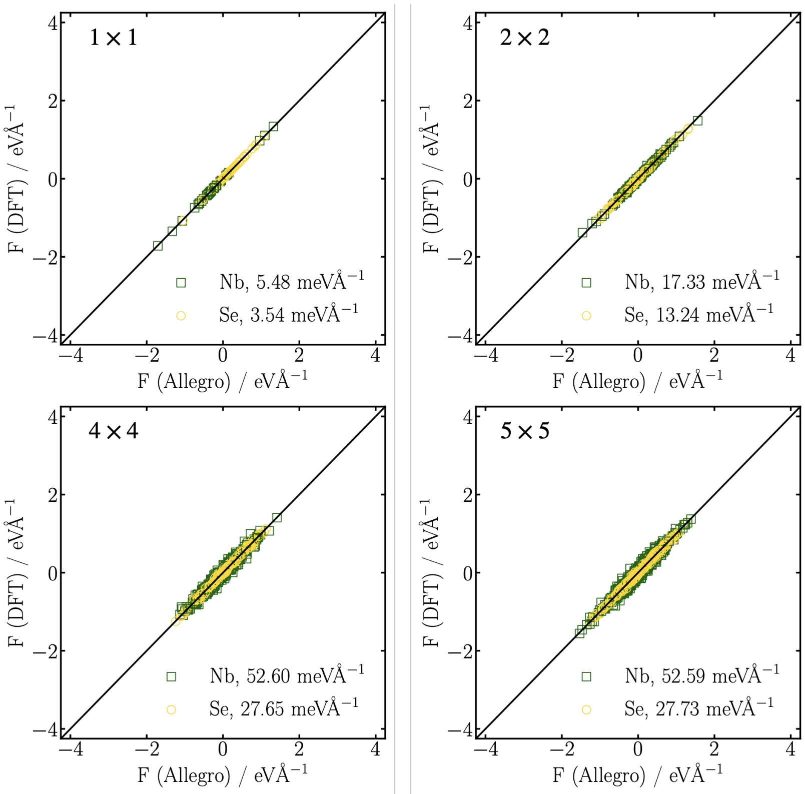

Figure S11: Force parity plots for the indicated structures from the **552** model after including incommensurate structures at 200 K, with MAEs as indicated.

In Fig. S19 we show the energy and force parity plots for the 3×3 cell at 200 K, which gave similar results to the **552** model in Fig. S6. Unlike the **552** model, the **1042** model gave the expected CDW structures on supercells of 3×3, i.e., the CDW structure was faithfully described with the correct period. In Fig. S20 and Fig. S21 we show, respectively, the force and energy parity plots of the **1042** model on incommensurate structures, as indicated. These perform worse than the **552** model, as seen in Figs. S8-S9 . Therefore, the **1042** model here also seems to generalize less well, as also found for the high-smearing case in Fig. S3.

We evaluated the performance of our refined Allegro model against the original **552** version, trained on the combined dataset of BAL and random-displacement relaxations, by comparing their phonon dispersions with DFT and DFPT calculations, as shown in Fig. S22. All finite-difference phonon calculations were performed with Phonopy using 3×3 supercells for both DFT and MLIP to ensure consistency and reduce computational cost. While this supercell size converges most of the spectrum, accurately describing the unstable soft mode requires larger cells as demonstrated by Bianco et al.[22] and discussed further below and in the main text.

In panel (a), the phonon dispersions predicted by both Allegro models are compared with DFT results. Both models reproduce the spectra well , though the refined model shows a slight reduction in accuracy on the 3×3 cell compared to the original. This tradeoff, already discussed in the main text, reflects the balance between optimizing for larger commensurate or incommensurate structures and retaining accuracy on small supercells. The refined model was ultimately preferred for its broader reliability across the relevant configuration space.

Panel (b) presents convergence tests with DFT phonons, which confirm that 3×3 and even 4×4 supercells are insufficient to achieve converged spectra. Instead, at least 6×6 commensurate cells are required within finite-difference calculations, and for full convergence a 12x12 is expected to be required. Panel (c) shows convergence tests with the refined Allegro model, which reveal an important limitation: the MLIP predictions are largely insensitive to the choice of supercell size used in the phonon calculation. This lack of transferability arises from two factors, as explained in the main text and expanded here. First, the cutoff radius r_{max} used during training corresponds to interactions within a 3×3 supercell (5 Å). Although one might expect the model to generalize to larger supercells, our results show this is not the case. Second, the training dataset itself is strongly biased toward 3×3 structures, which are insufficient for converged finite-difference phonons. The most promising strategy to address these limitations is therefore to refine the dataset by including larger supercells and to extend the real-space cutoff so that the model explicitly learns from

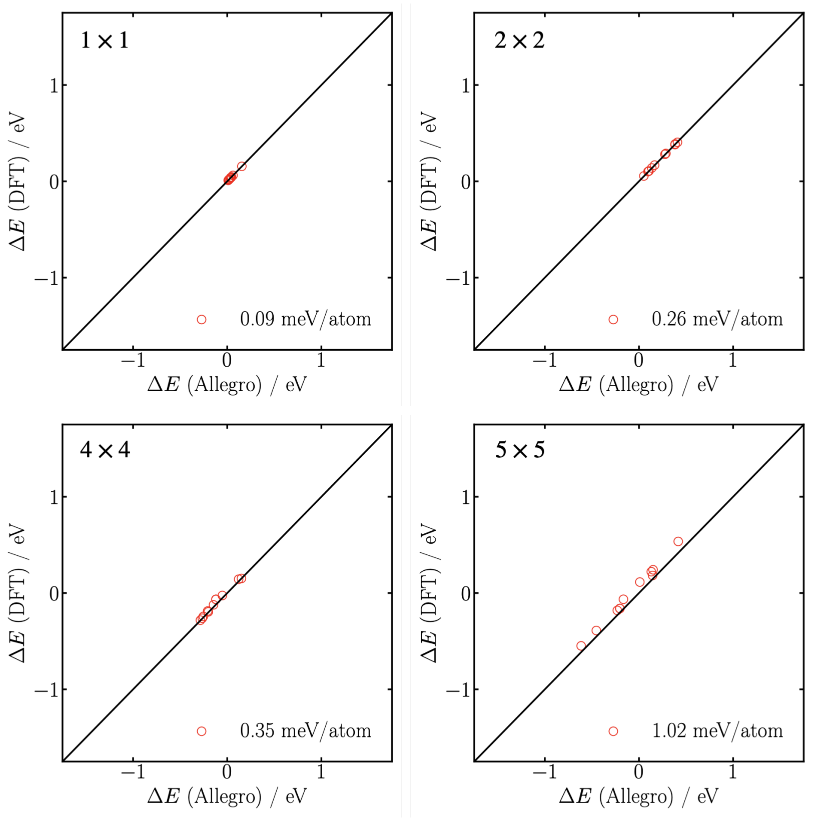

Figure S12: Energy parity plots for the indicated structures from the **552** model after including incommensurate structures at 200 K, with MAEs as indicated.

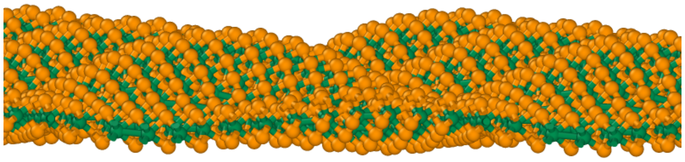

Figure S13: Example of rippling of the **552** model after including incommensurate structures when used for large supercells.

longer-range correlations. Finally, panel (d) shows the DFPT phonon dispersions, which highlight the target limit toward which finite-difference calculations, both with MLIP and DFT, should converge. In particular, the in-plane Nb–Nb displacement mode—central to the CDW transition—exhibits the expected double-well instability with its minimum at 2/3 along the Γ-M path, consistent with the 3×3 CDW phase and without spurious instabilities between M and K. These are precisely the spectral features we aim to reproduce. For DFPT, a 12×12 q-grid was used with a phonon self-consistency threshold of 1×10^{-20} Ry^2.

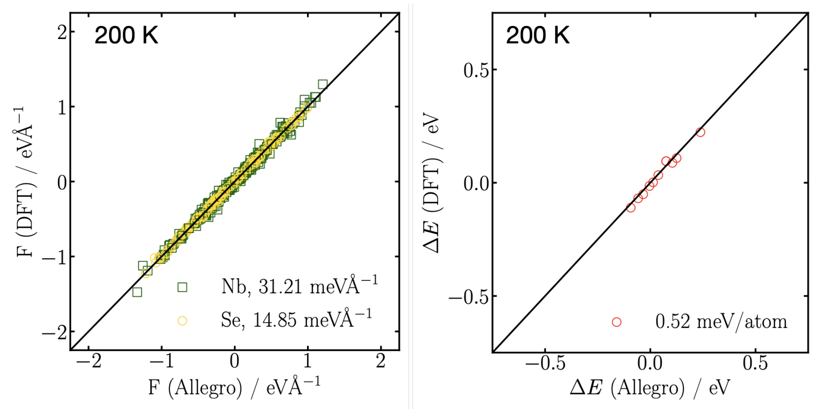

Figure S14: Force and energy parity plots at the indicated temperatures from the **552** model after including incommensurate and strained structures on a 3×3, with MAEs as indicated.

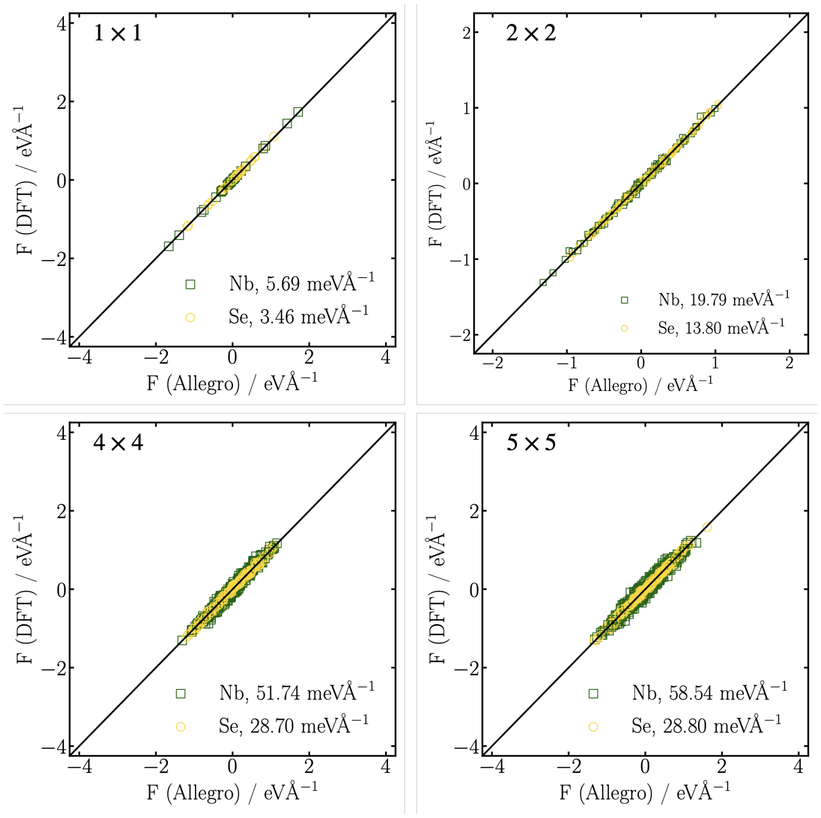

Figure S15: Force parity plots for the indicated structures from the **552** model after including incommensurate and strained structures at 200 K, with MAEs as indicated.

IV. ADDITIONAL RESULTS FOR MONOLAYER NBSE$_2$, WITH CDWS

A. Coexistence simulations for the hexagonal phase

The coexistence simulations discussed in the main text focus on competition between the two established triangular CDW phases (hollow and filled), for which the hollow phase is known to be energetically favored in the monolayer. Previous first-principles studies, however, have also reported the existence of an additional hexagonal CDW reconstruction that lies very close in energy to the undistorted structure and is therefore particularly challenging to stabilize. Motivated by these works, and to further test the ability of the MLIP to resolve subtle phase competition in shallow

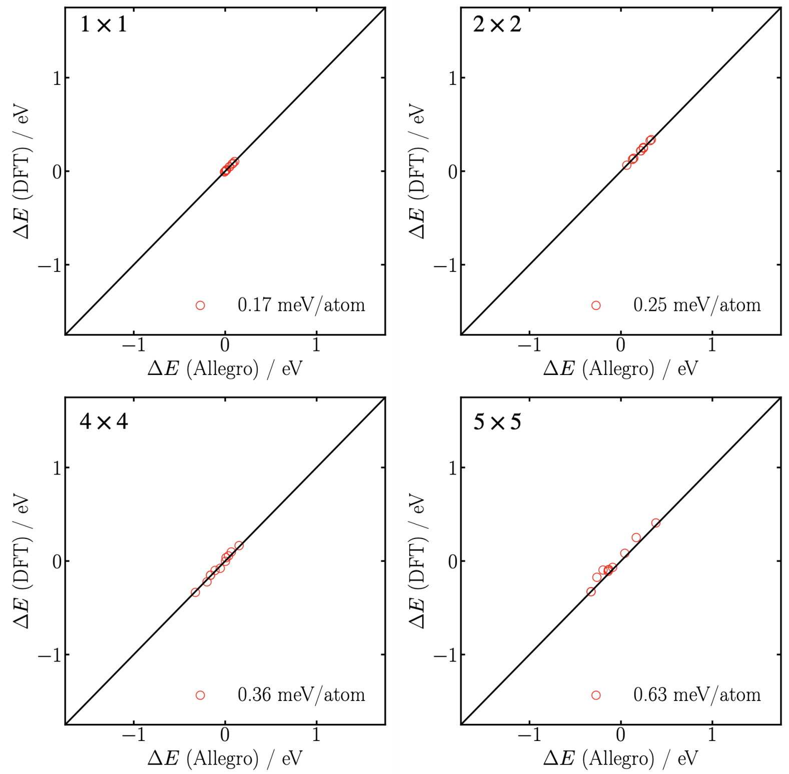

Figure S16: Energy parity plots for the indicated structures from the **552** model after including incommensurate and strained structures at 200 K, with MAEs as indicated.

energy landscapes, we extend the coexistence analysis to explicitly include hexagonal CDW motifs. We consider heterogeneous supercells containing normal, triangular (filled or hollow), and hexagonal CDW regions and examine their low-temperature dynamical evolution.

Figure S23 shows the resulting coexistence simulations for monolayer $NbSe_2$. Two initial configurations are considered, containing either filled–normal–hexagonal or hollow–normal–hexagonal CDW regions within the same 3×3 supercell. In both cases, the initially normal region rapidly develops CDW-like distortions, while the hexagonal CDW motif is unstable and progressively transforms into triangular CDW patterns. No long-lived hexagonal ordering is observed on the simulated timescale. Instead, the system evolves toward a uniform triangular CDW configuration whose character reflects the seeded triangular domain (filled or hollow). Compared to the bilayer case discussed later, the monolayer relaxes more rapidly and exhibits fewer residual defects, consistent with the simpler CDW energy landscape in the absence of interlayer coupling.

B. Coexistence simulations for incommensurate supercells

In Fig. S24 we display another example of a coexistence simulation for monolayer $NbSe_2$. This simulation is for a 12×12 structure, i.e., a 4×4 supercell of the CDW size. This simulation is initialized with the lower 2 rows of CDWs being in the hollow phase, while the top 2 rows of CDWs are initialized in the filled CDW phases. This structure can corresponds to $t = 0$ fs in Fig. S24. Similar to the monolayer case in the main text, we find that the hollow CDWs are more stable than the filled phases, and after 250 fs the lower row, which started in the filled phase, has a mixed hollow/filled structure, with the top row still being in the filled phase, albeit with some defects in the structure. After only 500 fs, the structure is almost entirely in the hollow phase, and it remains that way until the end of the simulation at 2000 fs.

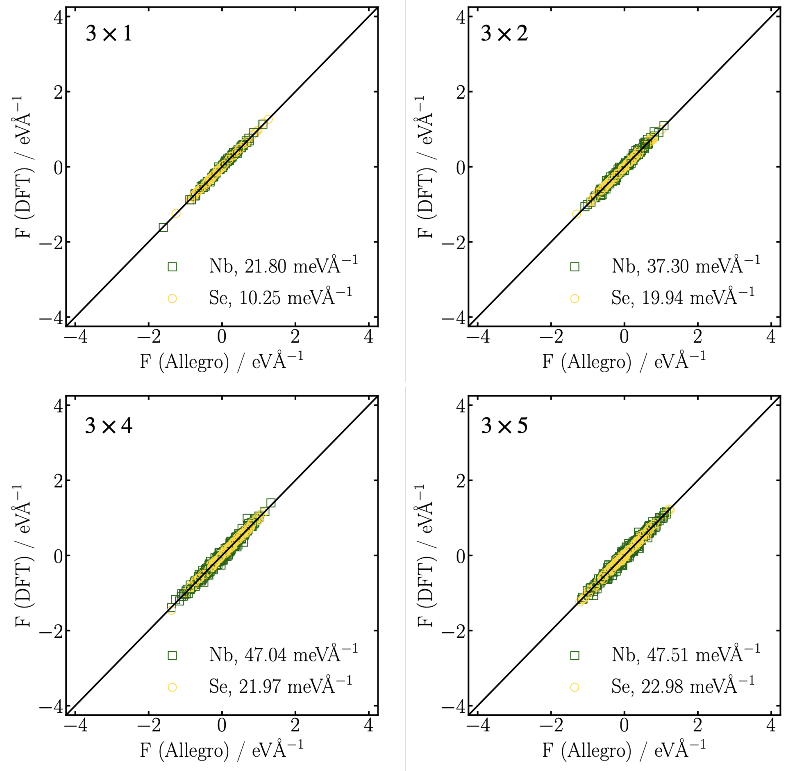

Figure S17: Force parity plots for the indicated structures from the **552** model after including incommensurate and strained structures at 200 K, with MAEs as indicated.

C. Commensurate-Incommensurate Structures

In Figs. S25-S26, we show MLIP predictions for incommensurate structures not trained on. Specifically, as indicated in the figures, for 3×4 to 3×8 in Fig. S25, and 3×10 to 3×14 in Fig. S26, missing any structures which are commensurate with the CDWs.

For 3×4, we find that there is coexistence of the hollow/filled CDW motifs, which tesselate together. For 3×5, the structure is more complex, and mainly resembles the filled CDWs. The 3×7, similar to the 3×4, has the coexistence of hollow/filled parts of CDWs, in addition to a fully formed filled phase. Whereas, the 3×8 mainly has the hollow phase, but there is a part of the incommensurate cell where there is a more complex structure.

Similarly, 3×10 cell is mainly filled, but with a more complex line pattern running thorough the cell. For 3×11, no clear CDW phase has dominated the structure, with coexistence of features which are present in hollow/filled structures. As these were just relaxations from pristine cells, it could be that for the larger structures some annealing is required. For 3×13, the hollow phase mainly exists in the incommensurate cell, with one of the hollow features being defective to accommodate the larger cell. Similarly, 3×14 is mainly hollow with a line defect running throughout the cell.

Overall, these predictions for incommensurate cells, *which are not trained on*, appear to be in line with what is expected, i.e., there is coexistence of the CDWs for smaller cells and larger cells mainly have one of the CDW phases with some features to accommodate the incommensurate cell.

D. Classical transition temperature estimates

In Fig. S27 we display our results for classical estimates of the transition temperatures from computing $g(r)$ for each element, at various different temperatures. As outlined in the Methods at the start of the SI, these were calculated from a 3×3 supercell of the CDW phase for the monolayer starting from random velocities initialized from the indicated starting structure at the temperatures shown. A short equilibration period of 10 ps was performed before the production run of 20 ps. The short equilibration was an attempt to keep some memory of the initialized phase, to see if there are any differences between hollow and filled in the monolayer, for example. Both Nb-Nb and Se-Se

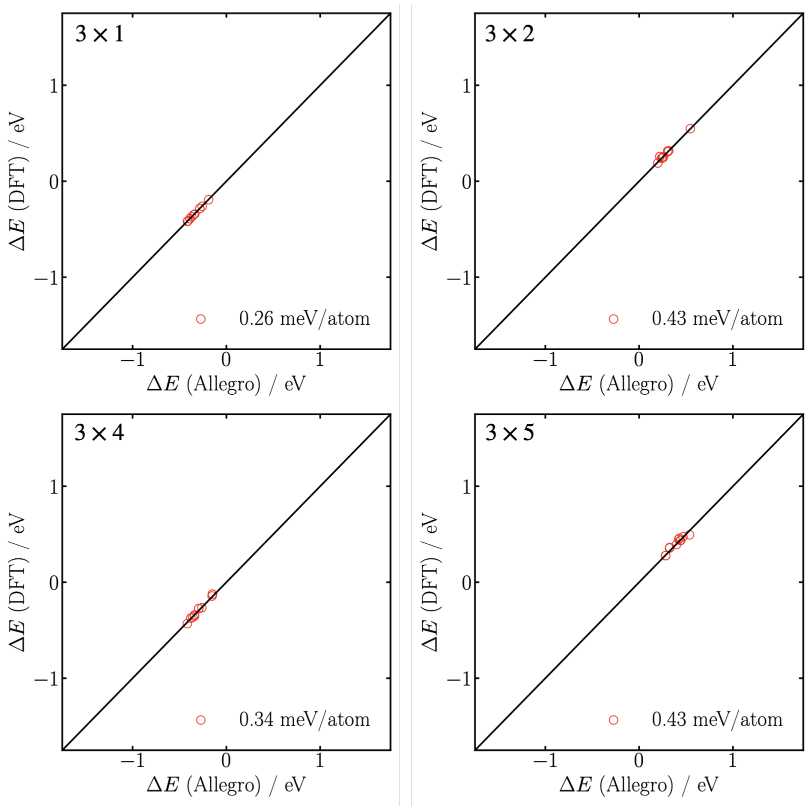

Figure S18: Energy parity plots for the indicated structures from the **552** model after including incommensurate and strained structures at 200 K, with MAEs as indicated.

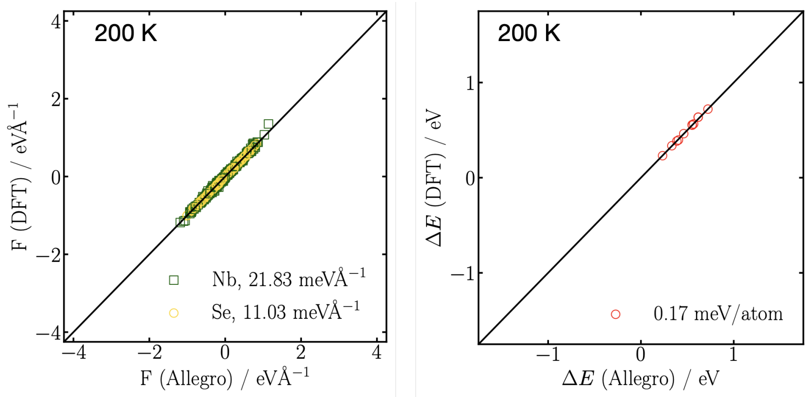

Figure S19: Force and energy parity plots at the indicated temperatures from the **1042** model after including incommensurate and strained structures on a 3 × 3, with MAEs as indicated.

$g(r)$ are indicated, with a gaussian fitted for each of these at every temperature.

For the monolayer starting in the hollow CDW phase, there are strong peaks in the $g(r)$ (for both Nb and Se) up to 20 K, with weak features all the way up to 50 K (mainly in the Nb).Similarly, starting from the filled phase, the monolayer $g(r)$ shows strong peaks up to 20 K, with deviations from the Gaussian fits remaining visible up to 40 K. Notably, the 1-20 K $g(r)$ between these phases are largely distinct, which could be used to characterize the phases. Upon inspecting the periodic lattice distortions of these phases, we found the filled CDW phase remained in the 1 K simulations, but in the 20 K simulations thermal energy was already large enough to convert the structure to the more stable hollow phase. For the structure initialized in the hollow phase, the periodic lattice distortions remained intact up to 20 K, but for larger temperatures, there is dynamic transitions between the different phases. To characterize this, and get a better estimate of the T_c from classical simulations, we further analyzed these simulations.

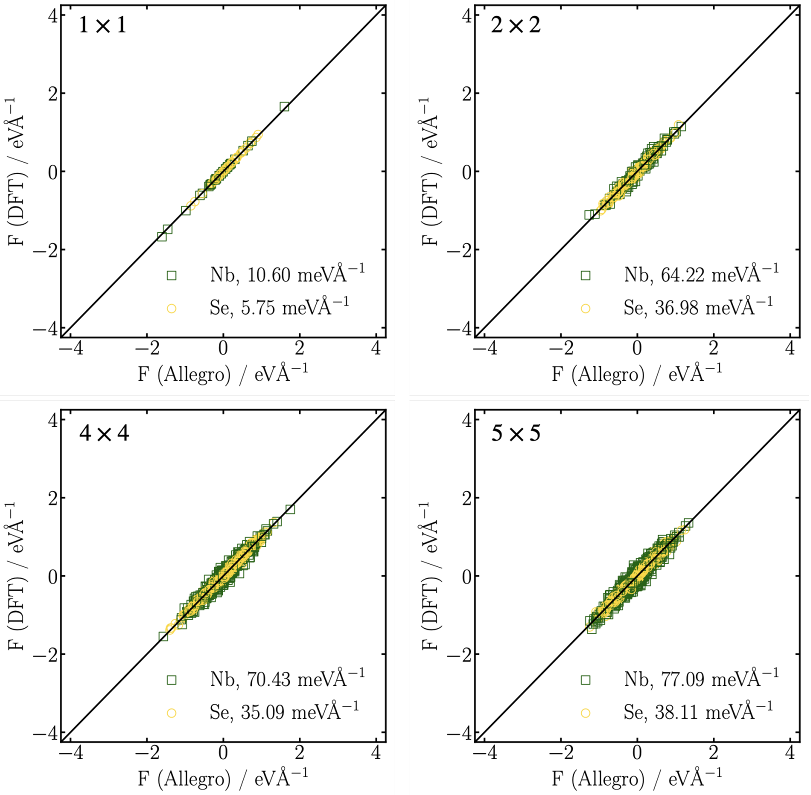

Figure S20: Force parity plots for the indicated structures from the **1042** model after including incommensurate and strained structures at 200 K, with MAEs as indicated.

In Figs. S28 and S29, we show the values of the order parameters of each phase, computed from the method outlined in Ref. [23], as a function of time, for the indicated temperature, when the structure was initialized in a hollow and filled phase, respectively. Starting from the hollow phase, we see the order parameter remains at 1 for 1 K, indicating a perfect phase. For 10 K already, there are some deviations from the ideal phase, owing to thermal fluctuations. At 20 K and 30 K, there has been a substantial suppression in the order parameter of the phases, but it remains to be distinctly in the hollow phase. For larger temperatures, as seen in the 40-50 K panels, interconversion between the hollow and filled phase is observed, but the order parameter for these remains low.

In contrast, for the calculations initialized in the filled phase, even at 1 K, there are deviations of the periodic lattice distortions from the ideal one. Moreover at 10 K, the order parameter of the filled phase is strongly suppressed. At 20 K already, after the equilibration period, the CDW phase has converted to the lower-energy hollow phase. For larger temperatures, the order parameter values then resemble those that were initialized in the hollow phase.

From these observations, we estimate the transition temperature of the hollow phase to be around 40 K, while that of the filled phase lies near 10 K.For the hollow phase, this conclusion was drawn from the $g(r)$ still having slight discrepancies from a gaussian fit at 40 K, the hollow phase periodic lattice distortions clearly being observed in the structures, and the order parameter of the hollow phase being larger than 1/4. The transition temperature for the filled phase was mainly drawn from the observations of the structures and calculations of the order parameters, since it converts to the more stable hollow phase at larger temperatures.

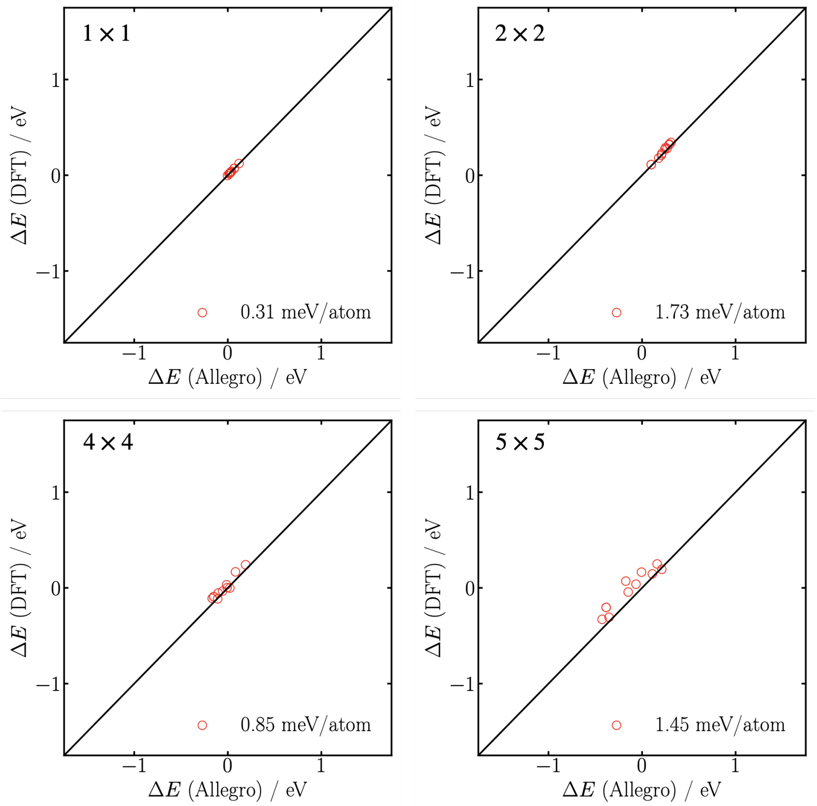

Figure S21: Energy parity plots for the indicated structures from the **1042** model after including incommensurate and strained structures at 200 K, with MAEs as indicated.

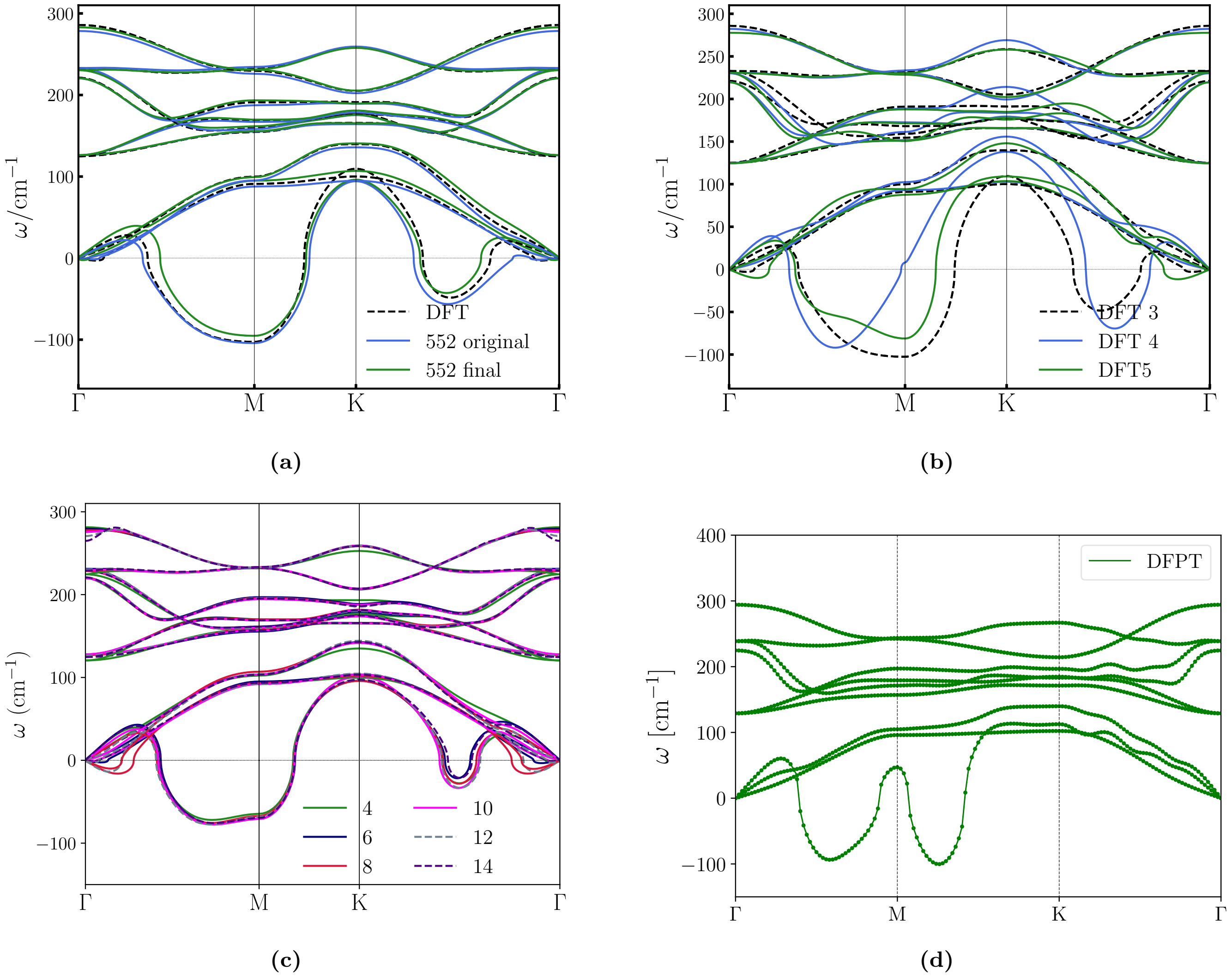

Figure S22: Phonon dispersion for the normal phase of the monolayer; comparisons between DFT, DFPT, and MLIP models. (a) Phonon dispersions from DFT and both Allegro models (original and refined) using finite differences with a 3×3 supercell. (b) Convergence test of DFT phonon dispersions with respect to the supercell size. (c) Supercell size convergence for the refined Allegro model, highlighting the need for larger cells (at least 6×6) to accurately capture the unstable phonon mode. (d) DFPT phonon dispersions, showing qualitative differences from finite-difference results.

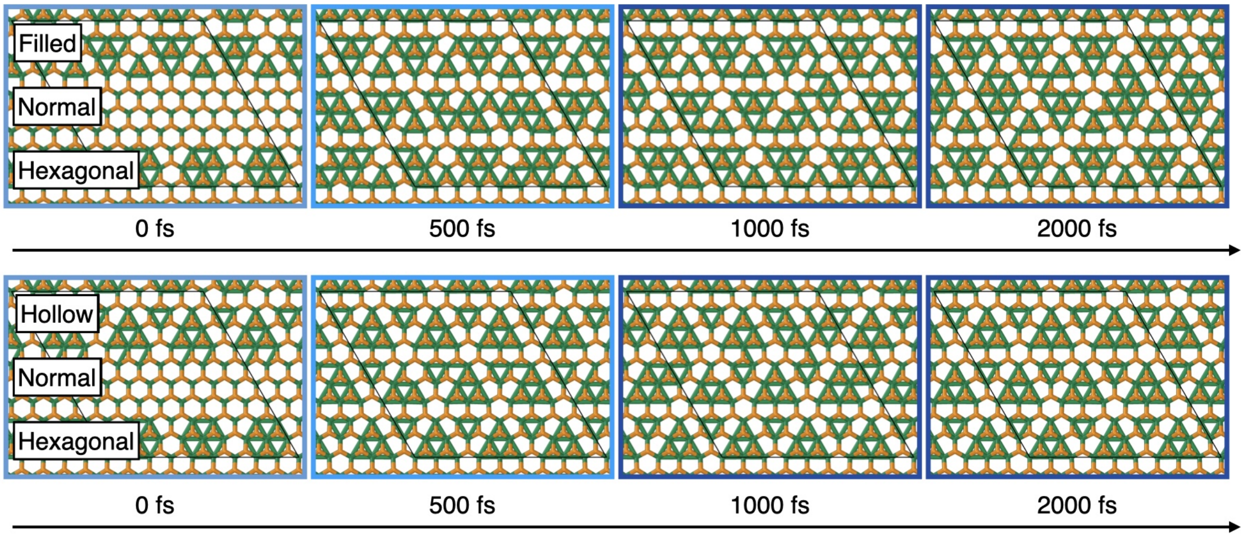

Figure S23: Allegro predictions for the evolution of coexisting CDW phases in a monolayer $NbSe_2$ 3×3 supercell at 10 K. Two heterogeneous initial configurations are shown: the top row starts from coexisting filled, normal, and hexagonal CDW regions, while the bottom row starts from coexisting hollow, normal, and hexagonal CDW regions.

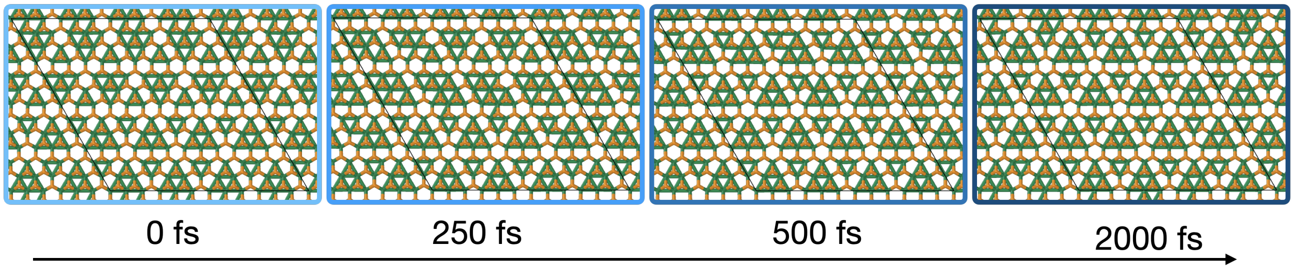

Figure S24: Allegro predictions for the evolution of coexisting CDW phases in a 4×4 supercell of the CDW. The monolayer starts from a coexisting structure of filled/hollow, with half being filled and the other half being hollow.

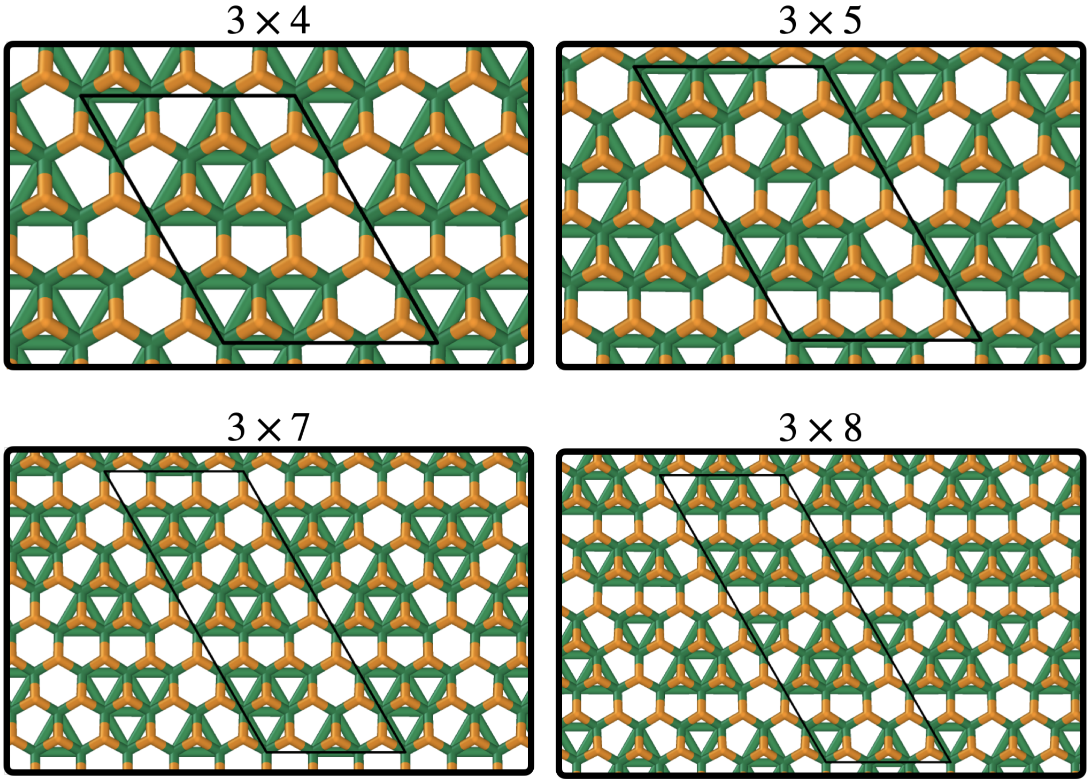

Figure S25: Allegro predictions for commensurate-incommensurate structures, as indicated. .

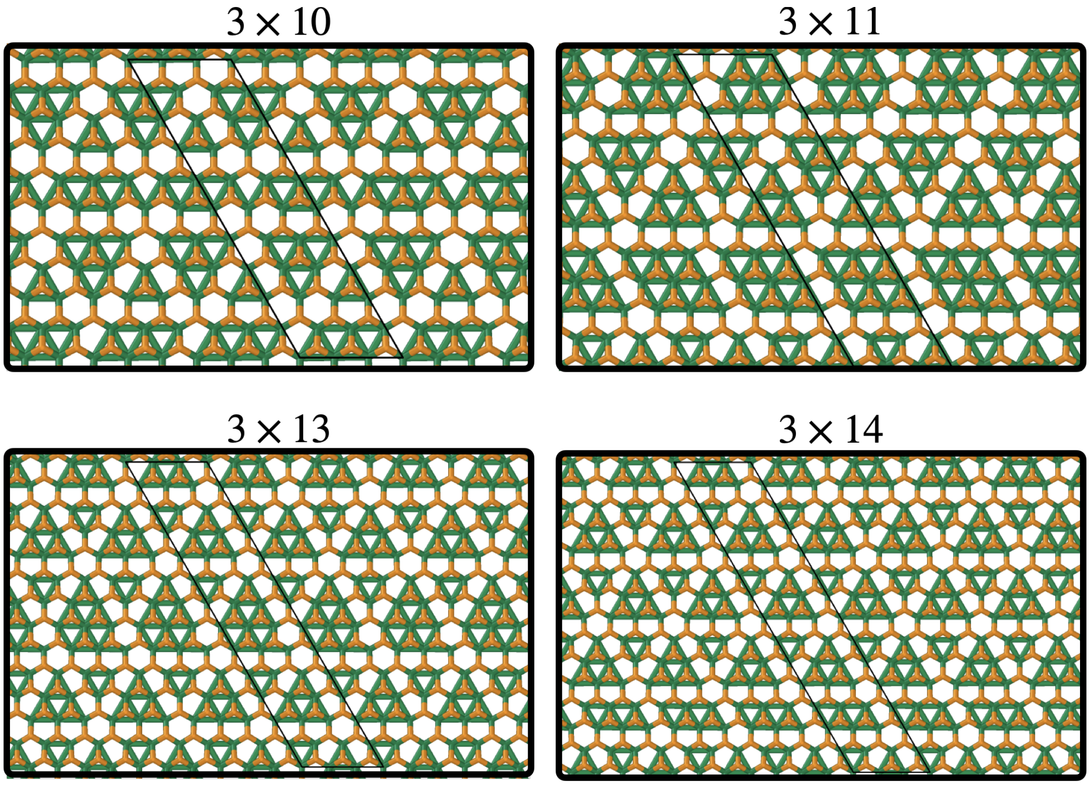

Figure S26: Allegro predictions for commensurate-incommensurate structures, as indicated.

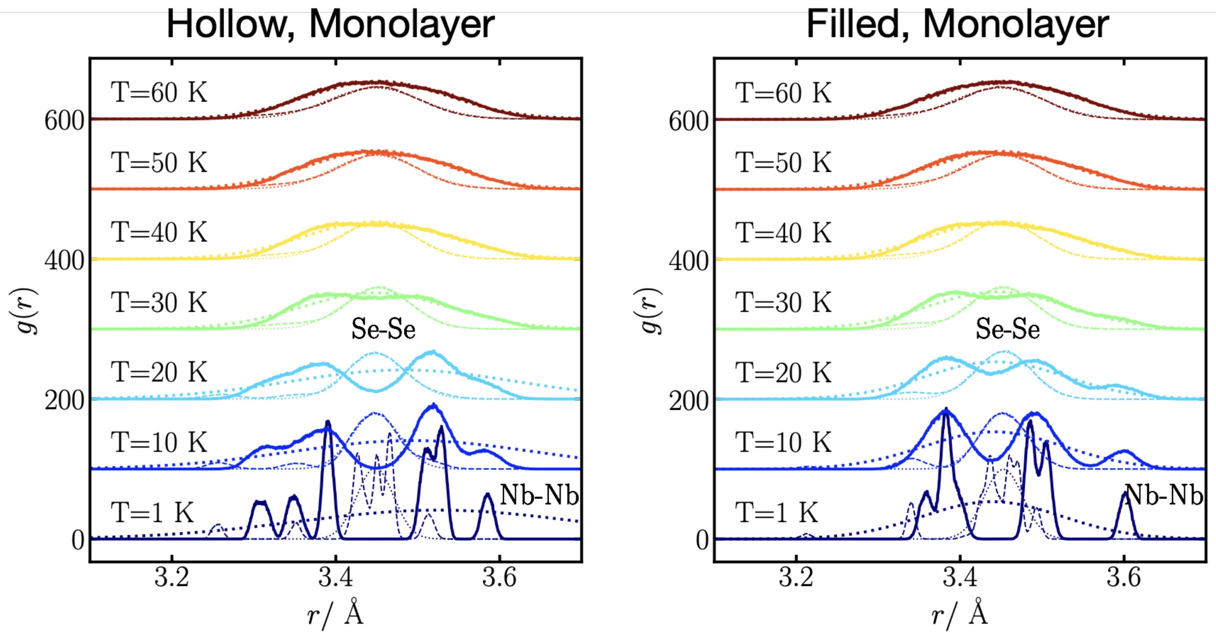

Figure S27: Element resolved $g(r)$ as a function of separation for the indicated temperatures and starting structures.

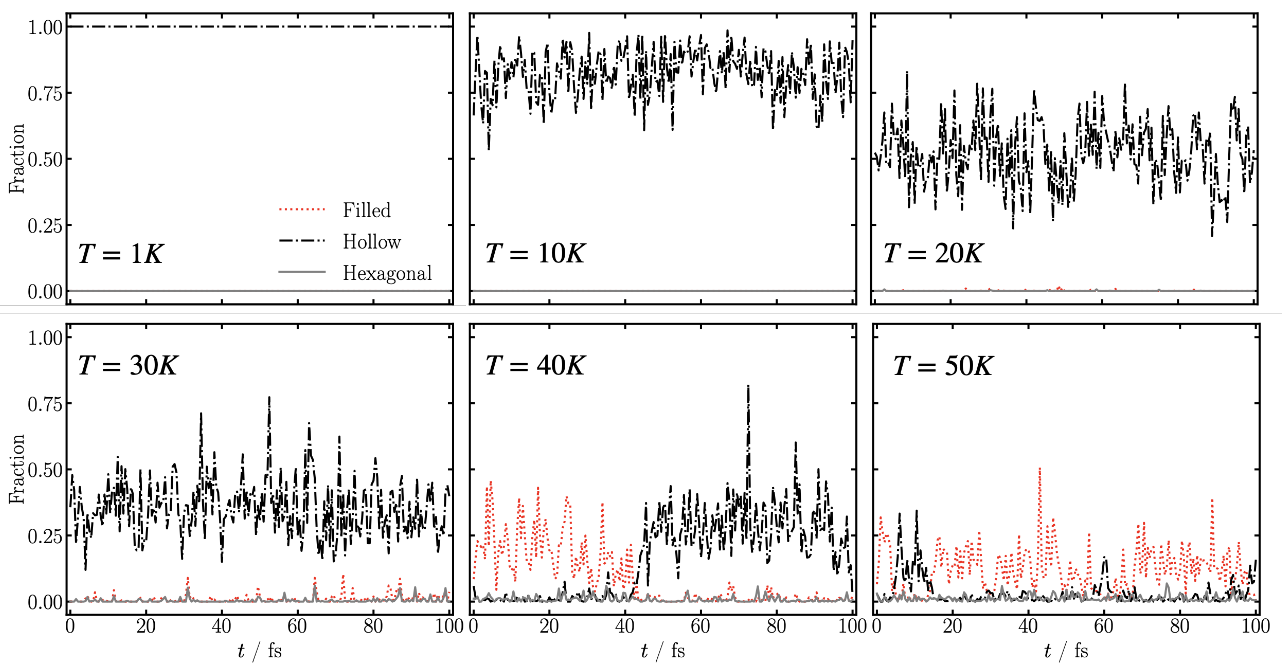

Figure S28: Order parameter of the CDW phases, calculated from the method in Ref. 23, as a function of time and for a number of temperatures, starting from the hollow phase.

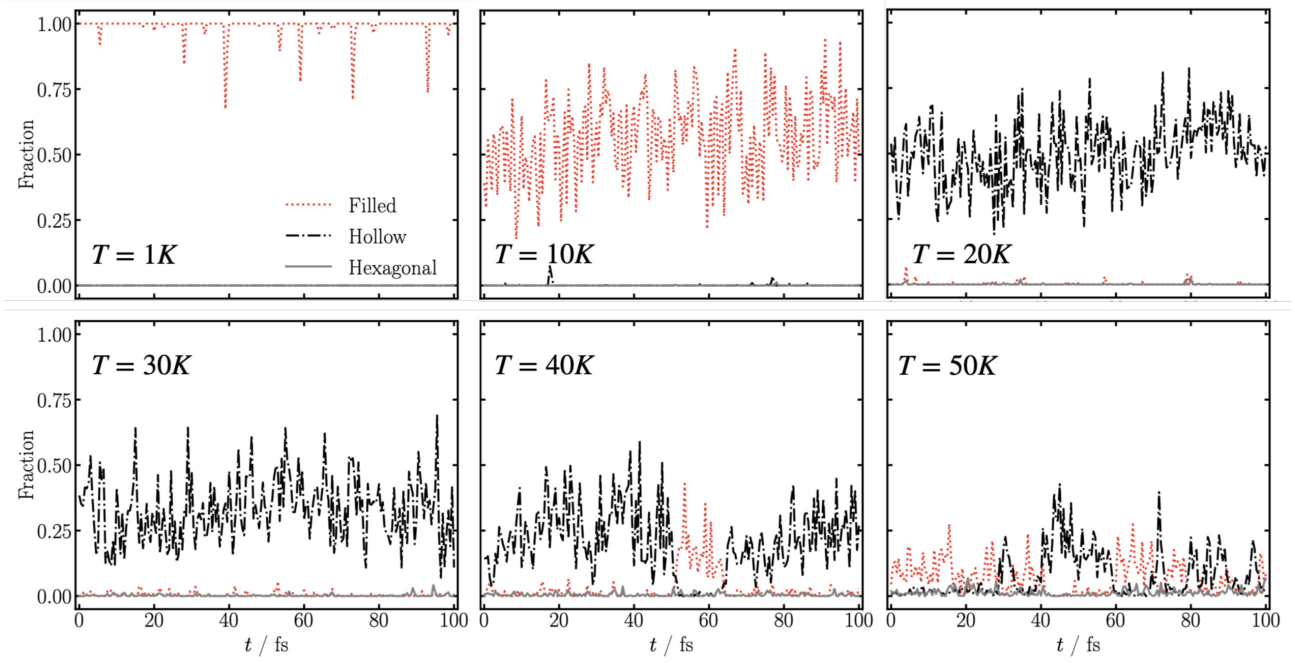

Figure S29: Order parameter of the CDW phases, calculated from the method in Ref. 23, as a function of time and for a number of temperatures, starting from the filled phase.

V. BILAYER NBSE$_2$, WITH CDWS

In this section, we outline the training procedure and hyperparameter scans conducted using the final dataset to model bilayer $NbSe_2$ (with CDW phases). We begin with a hyperparameter scan on a dataset of 1484 structures, followed by tests similar to those previously performed for the monolayers.

A. Hyperparameter scan

In Table S9, we present the validation errors for training Allegro models on a dataset of 1484 structures (referred to as the BAL dataset for monolayers and bilayers, with bilayers originating from five distinct stackings and both parallel and antiparallel alignments). The errors are evaluated by varying r_{max} for different values of l_{max}, with the parameter choices based on the monolayer hyperparameter scan, while keeping all other hyperparameters fixed. The improvement given by choosing l_{max}=5 over 4 is not significant, thus we decide to focus on l_{max}=4 and in the following we compare the tests performed for two models: **552** and **842**.

**Model**	F (Nb) / meVÅ^{-1}	F (Se) / meVÅ^{-1}	E / meV/atom
**542**	28.07	15.51	1.41
**742**	23.77	13.23	0.155
**842**	**23.02**	**12.35**	**0.138**
**552**	28.15	14.97	0.403
**752**	23.42	12.07	**0.132**
**852**	**22.23**	**12.05**	0.145

Table S9: Bilayer validation errors from combined dataset BAL for both monolayer and bilayers.

B. Test

We extensively tested the **552** and **842** models by performing MD simulations at 200 K to collect structures for the 3×3 supercell, along with their respective energy and force predictions. These predictions were then compared with the ground-truth results from DFT. In Fig. S30, we show the resulting force and energy parity plots (MAEs) for the **552** model; the corresponding plots for the **842** model are shown in the main text, and we won't repeat them here. As can be seen, good errors were also obtained for the **552** model, comparable to validation errors and those of the **842** model. In fact, the **552** model has slightly better energy errors, but slightly worse force errors.

As a second check, we compared the energy binding curves against DFT results for different stackings. The results for the **842** model are presented in the main text (Fig. 4); here, for completeness, we also report the results for the **552** model. As shown in Fig. S31, the **552** model describes the low-energy binding curves worse than the **842** model, although the **552** model appears to extrapolate well to the large-energy parts of the binding curves, better than the **842** model. As $r_{\mathrm{max}} = 5$ Å is too small to capture contributions from Nb–Nb interlayer environments, given that the equilibrium interlayer separation is between 6 and 7 Å, it is not surprising that the **842** model reproduces the most relevant parts of the binding energy curves.

Lastly, we focus on the **842** model and compare the phonon dispersions obtained from this model with DFT, both computed using finite-difference methods, as reported in the main text (Fig. 4), using 3×3 supercell (note that DFT phonon calculations for bilayers are relatively computationally expensive). This choice serves to demonstrate good agreement between our MLIP and DFT, provided that the supercell size is consistent. However, since we aim to capture subtle differences in phonon energies and the detailed characteristics of the soft modes, we found that (consistent with previous observations for the monolayer) accurately locating the minimum of this mode requires at least a 4×4 supercell, and preferably a 6×6 or larger one, to reproduce the full unstable mode and suppress spurious instabilities. This is shown in Fig. S32 (a) for the natural stacking and (b) for the AB stacking. This is particularly important for the subsequent estimation of the critical temperature. We emphasize that a 4×4 supercell is sufficient for most purposes, but a 6×6 supercell is necessary for more accurate convergence. Similar checks were also performed for the AA stacking, and the same considerations apply.

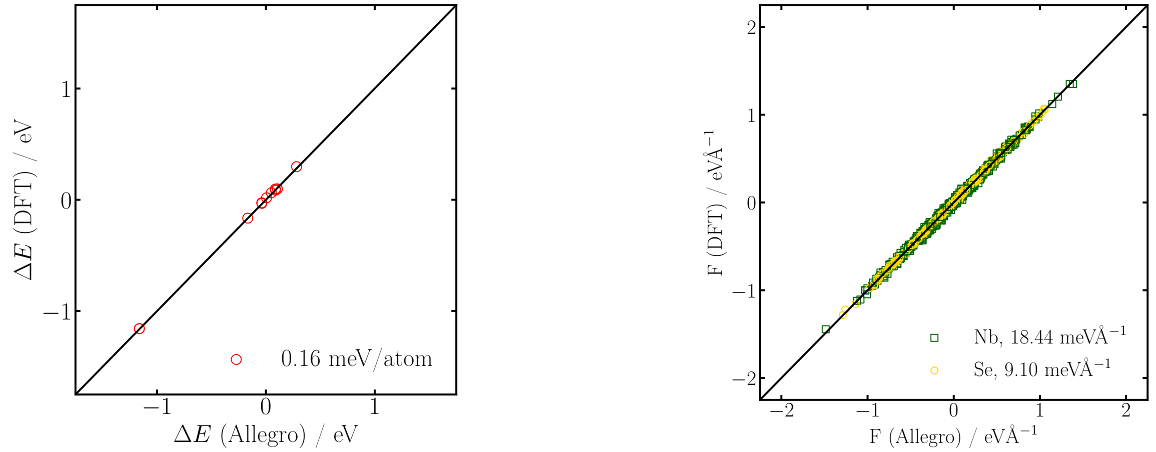

(a) (b)

Figure S30: Energy (a) and force (b) parity plots for bilayer $NbSe_2$ at 200 K for the **552** model, with MAEs as indicated.

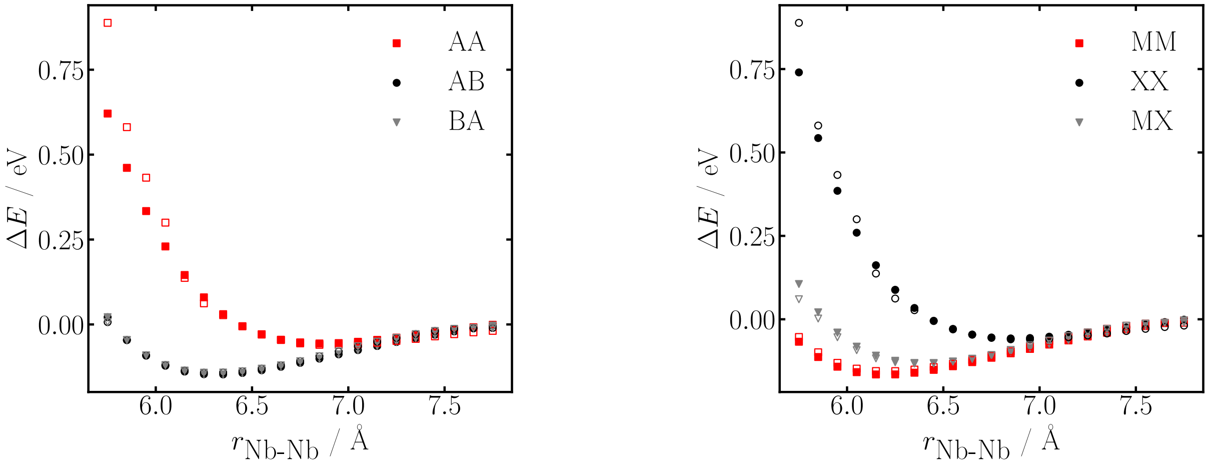

(a) (b)

Figure S31: Binding energy curves for the indicated stackings at 0°(a) and 180°(b) for the **552** bilayer model. The energy change is not normalized by the number of atoms, and is set to 0 for the largest separation studied. These were calculated with 1×1 cells. Filled symbols represent Allegro MLIP results, and empty symbols represent DFT results.

VI. ADDITIONAL RESULTS FOR BILAYERS NBSE$_2$

A. Coexistence simulations for the AB Bilayer

In Fig. S33 we display the results for the coexistence simulation for the AB bilayer $NbSe_2$ at 10 K. We start the simulation from a CDW structure in each layer that comprises of a strip of the filled phase, normal, and hollow, which approximately reside on top of each other in the bilayer. This initial structure can be seen in the left panels of Fig. S33 at $t = 0$ fs, with the top layer being shown in the top panels, and the bottom layer being shown at the bottom. At 500 fs, the part of the structure which was originally in the normal phase has been populated with CDW-like structures that resemble hollow/filled CDWs. At 2000 fs, the top layer has almost entirely converted to the hollow phase, while the bottom layer is mainly filled, but significant defects in the CDW structure exist. After 4000 fs, the top layer is relatively stable in the hollow phase, and the bottom layer is largely the filled phase, but the CDW structure in this bottom filled layer is significantly more dynamic.

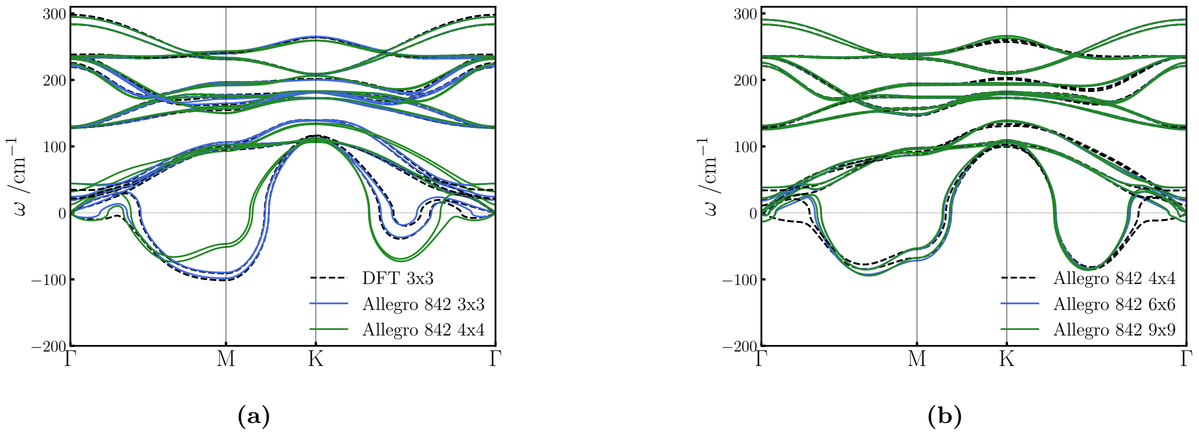

Figure S32: **a** - Phonon dispersion comparison for the MM natural stacking, showing results from DFT and Allegro 842 calculations. The 3×3 supercell is compared against the 4×4 supercell for the Allegro 842 model. **b** Comparison of the Allegro 842 model for AB stacking structures, evaluated across different supercell sizes.

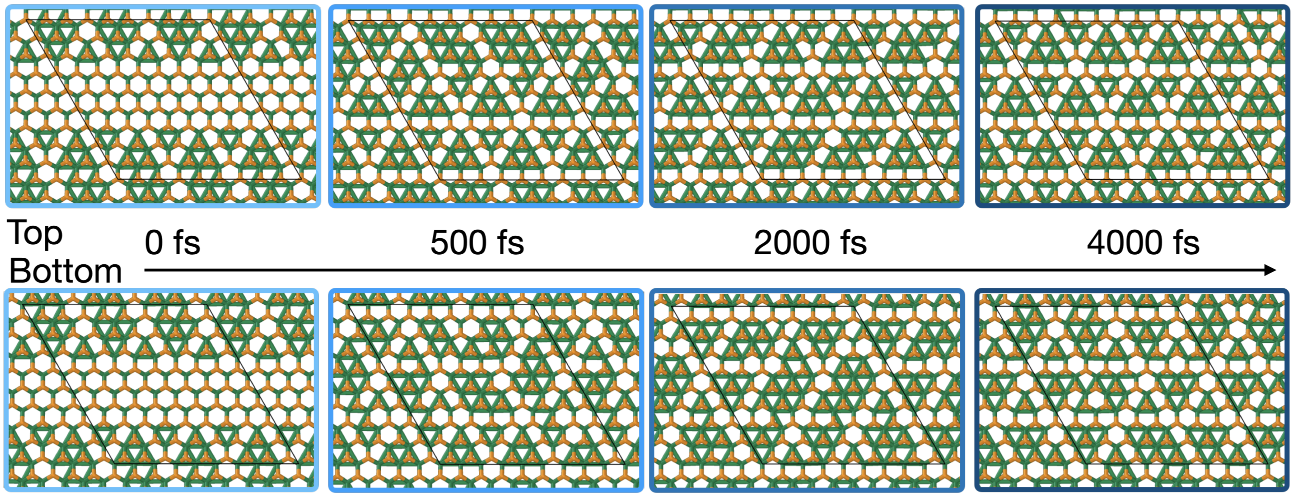

Figure S33: Allegro predictions for the evolution of the CDW phases in an AB bilayer for a 3×3 supercell of the CDW phases at 10 K. Each layer starts from a coexisting structure of filled/normal/hollow, with each of these phases in each layer approximately residing on top of each other.

B. Coexistence simulations with the hexagonal phase for the natural stacking

Following the same coexistence protocol described above for the AB bilayer, we performed an additional low-temperature simulation for the naturally stacked bilayer in which filled, normal, and hexagonal CDW regions were explicitly seeded within the same supercell. Starting from this heterogeneous configuration, we ran NVT molecular dynamics at 10 K and monitored the structural evolution of both layers over several picoseconds (Fig. S34). We observe that the initially normal region rapidly develops CDW-like distortions, while the hexagonal CDW motif is unstable and progressively transforms into triangular CDW patterns. Within approximately 1 ps, the system evolves toward a configuration dominated by triangular CDWs, and no persistent hexagonal ordering is observed on the simulated timescale. This behavior indicates that, under these conditions, the hexagonal CDW does not correspond to a long-lived metastable minimum, but instead represents a shallow and dynamically unstable configuration. These results are consistent with previous first-principles studies identifying the hexagonal CDW as energetically disfavored in pristine $NbSe_2$, and further support the ability of the MLIP to reproduce the correct hierarchy of competing CDW phases. We note that the resulting triangular CDW configuration is predominantly of filled character and remains dynamically trapped on the simulated timescale, exhibiting fluctuations around this metastable state rather than relaxing fully to the hollow CDW ground state. This behavior is consistent with the extremely small energy

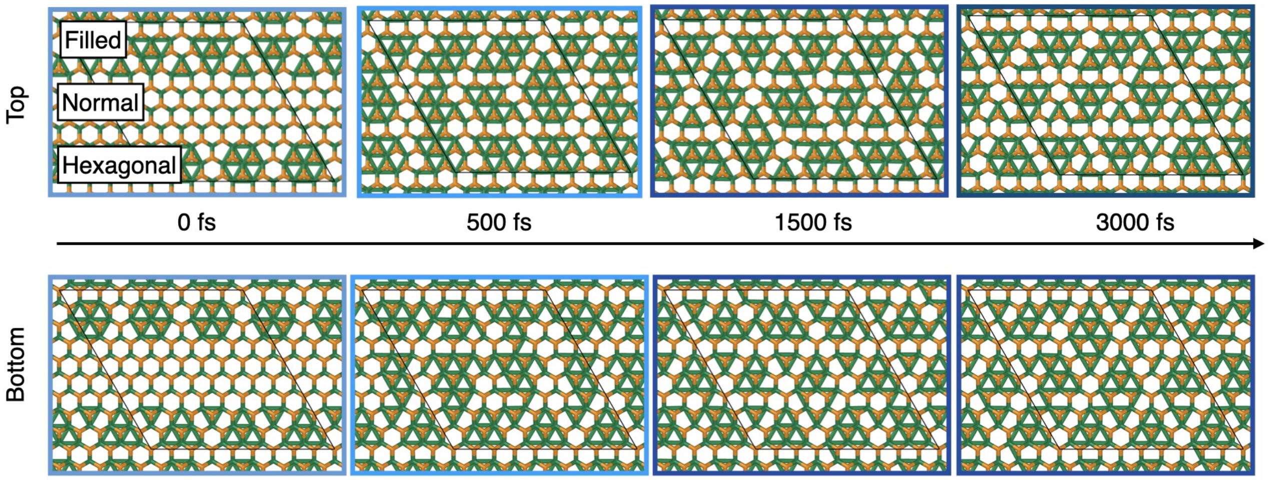

Figure S34: Allegro predictions for the evolution of the CDW phases in a naturally stacked $NbSe_2$ for a 3×3 supercell of the CDW phases at 10 K. Each layer starts from a coexisting structure of filled/normal/hexagonal, with each of these phases in each layer approximately residing on top of each other.

separation between filled and hollow CDW phases and the limited timescales accessible to low-temperature molecular dynamics, for which kinetic trapping in shallow metastable minima is expected.

We next consider a coexistence simulation initialized from hollow, normal, and hexagonal CDW regions in a naturally stacked $NbSe_2$ bilayer (Fig. S35). Starting from this heterogeneous configuration, the initially normal region rapidly develops CDW-like distortions, while the hexagonal CDW motif proves unstable and progressively transforms into triangular patterns. On the simulated timescale, the system evolves toward a mixed triangular configuration in which one layer predominantly adopts a well-ordered hollow-type CDW, while the other layer exhibits a filled-type CDW with increased defects and dynamical fluctuations. No persistent hexagonal ordering is observed at long times. The higher degree of spatial coherence in the hollow layer compared to the filled one is consistent with the greater intrinsic stability of the hollow CDW phase, while the continued fluctuations in the filled layer reflect its metastable character in the bilayer.

We further note that the relaxation dynamics in the bilayer are significantly slower than in the corresponding monolayer coexistence simulations, and that initializing the system from a heterogeneous configuration containing the hexagonal CDW motif further prolongs the relaxation time compared to the hollow–normal–filled coexistence cases discussed in the main text. This reflects the increased complexity of the bilayer CDW energy landscape, where competing intra- and interlayer distortions give rise to multiple shallow minima and enhanced kinetic trapping. As a result, on the simulated timescales the system may remain trapped in metastable triangular CDW configurations—most prominently of filled character in this case—while still correctly reproducing the instability of the hexagonal phase and the hierarchy among competing CDW motifs.

C. Stacking of CDWs

In Fig. S36, we have tabulated visually the unique stacking orders of the CDWs phases in the MM stacking configuration. In total, we found 3 unique stacking arrangements for the hollow-hollow phases in each layer, 4 unique stackings for the filled-hollow (and equivalently the hollow-filled), and 3 for stacking filled-filled. Overall, we find that there are very small energy differences between these different stacking orders of the CDWs, with only $\sim$ 10 meV between them in DFT, corresponding to an energy difference between them of 0.2 meV/atom. The lowest energy stackings from DFT (-77 meV relative to the reference energy, E_0, taken to be -50159.6 eV) is found to be the with the filled-hollow phases, with 2 structures being degenerate in energy. While the Allegro MLIP finds one of these to be the lowest energy (-49 meV relative to the reference energy, E_0), and the other degenerate stacking in DFT to be the second most stable, there are clear deviations between the ground-truth DFT data and the CDW stacking energy landscape in the Allegro MLIP. Moreover, there is an apparent constant shift between the DFT labels and the values predicted by the Allegro model, which corresponds to an error of approximately 0.4 meV/atom, which is of the order found upon testing the model previously. Therefore, we have demonstrated that our Allegro MLIP can qualitatively

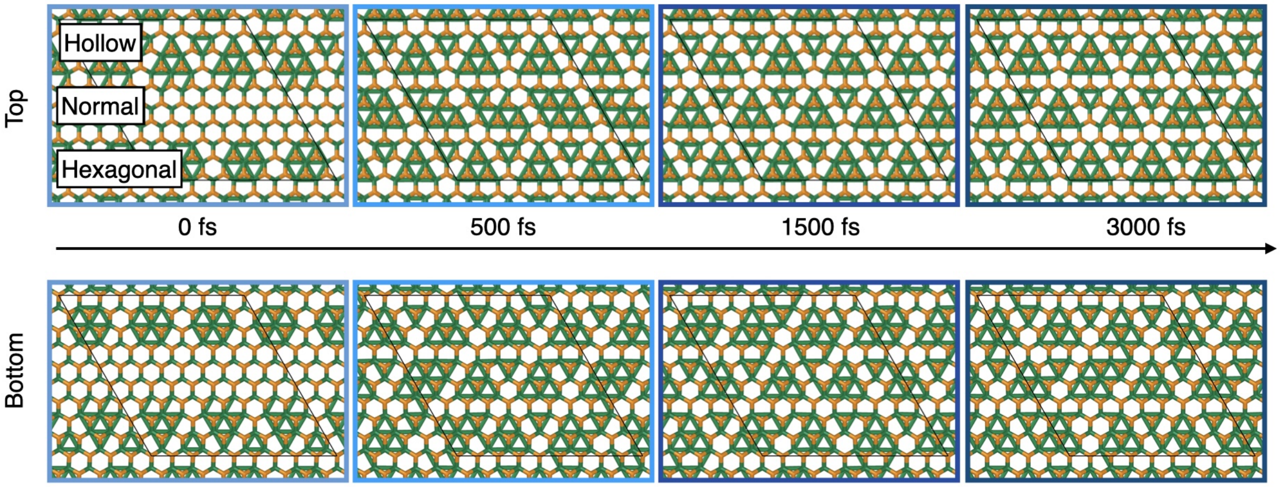

Figure S35: Allegro predictions for the evolution of the CDW phases in a naturally stacked $NbSe_2$ for a 3×3 supercell of the CDW phases at 10 K. Each layer starts from a coexisting structure of hollow/normal/hexagonal, with each of these phases in each layer approximately residing on top of each other.

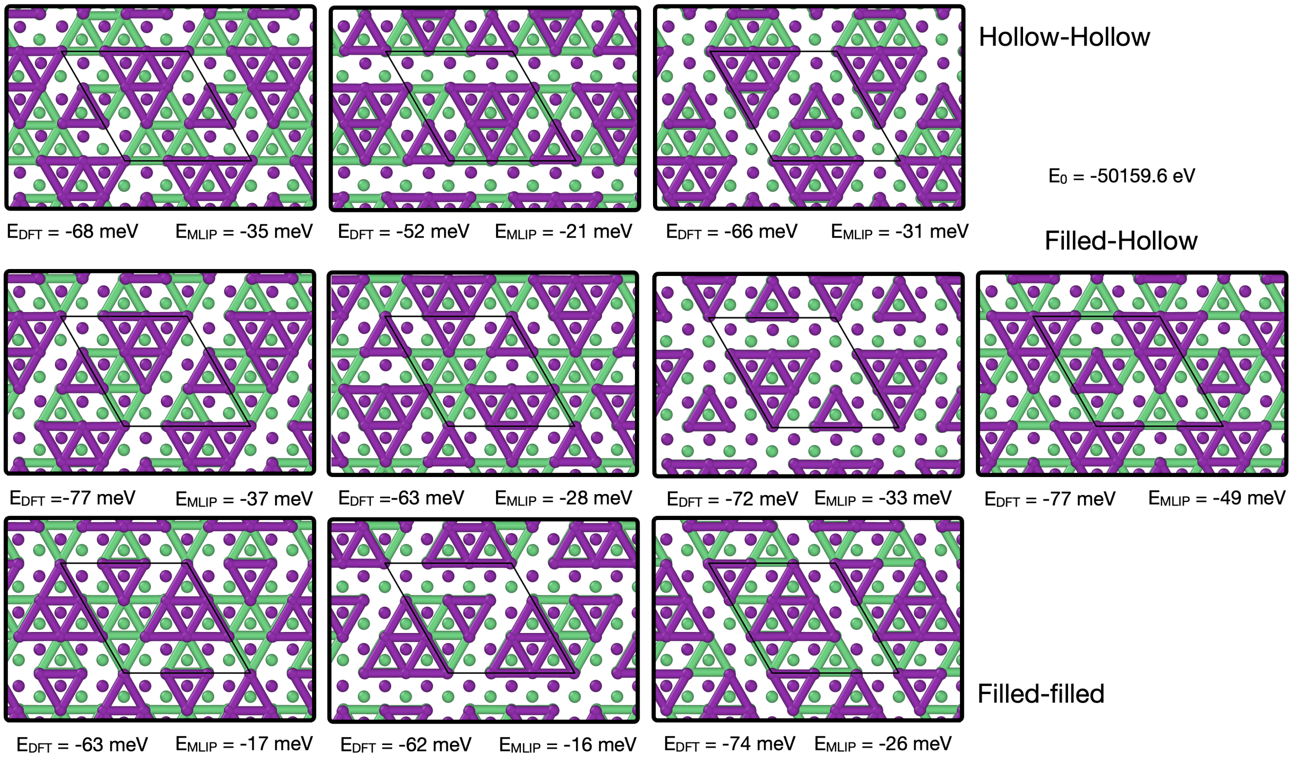

Figure S36: Structure and energy, from DFT and Allegro, for the unique stacking orders of the CDWs for the MM stacking.

find the correct CDW phases to be hollow-filled, it struggles with the exact ordering of all the possible stackings of the CDWs.

Similarly in Fig. S37 we show the unique CDW stackings for the AB bilayer configuration, where we again find an extremely flat energy landscape. From DFT, we consistently find the holllow-hollow CDW stacking to be the lowest in energy, with the others being very close in energy. We again find that the Allegro MLIP can qualitatively match the DFT, with hollow-hollow again being the lowest energy, but there are difficulties in the exact energies and ordering

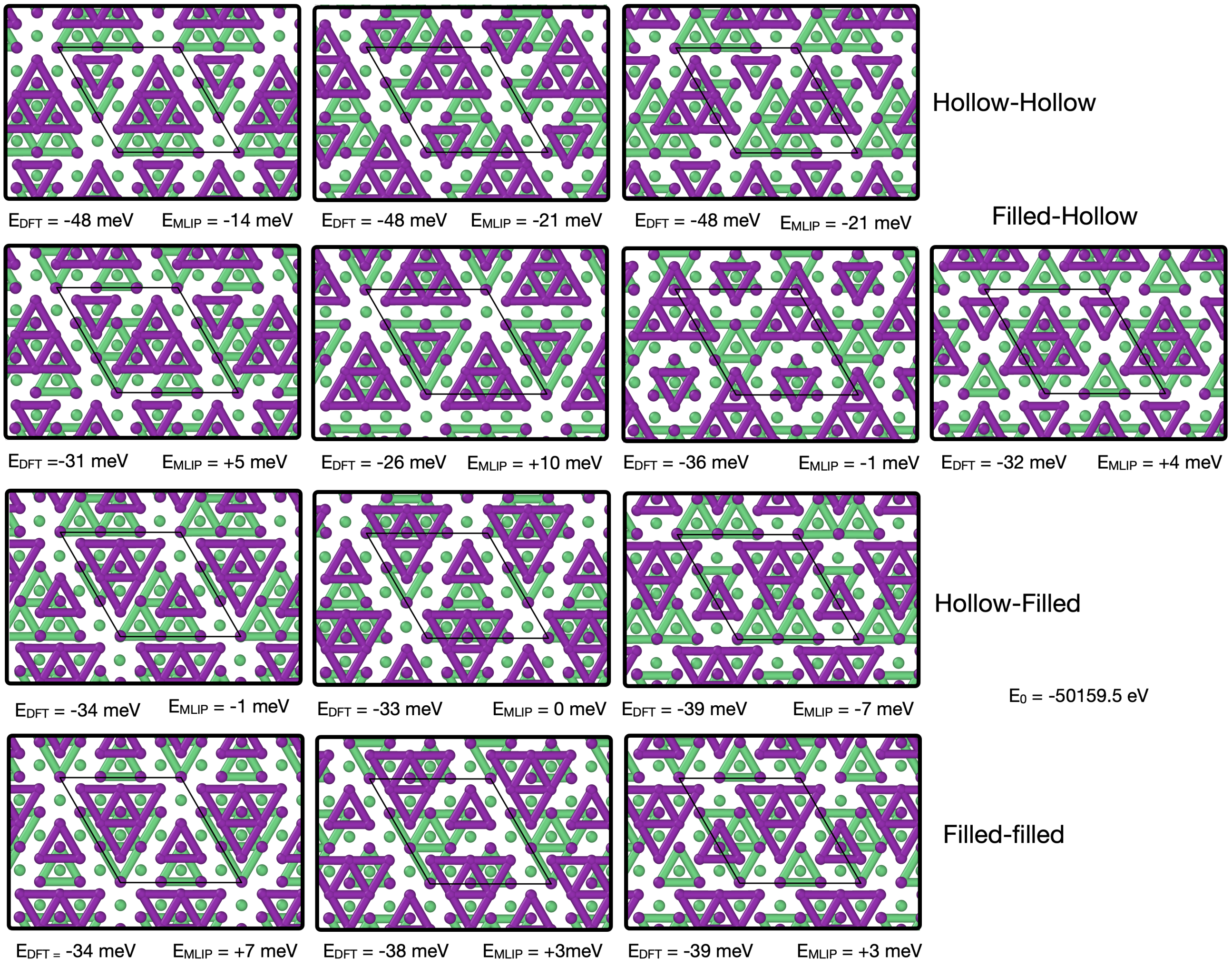

Figure S37: Structure and energy, from DFT and Allegro, for the unique stacking orders of the CDWs for the AB stacking.

of the different hollow-hollow configurations, in addition to the other stackings of different CDW phases. The energy error for the AB bilayer appears to be closer to 30 mev, which corresponds to 0.55 meV/atom.

Overall, we find our Allegro MLIP can qualitatively match the energy landscape of the most stable CDW phase in each layer, but that it struggles quantitatively with the different energies of how these CDWs stack on top of each other. This appears to be a subtle problem to capture with MLIPs, however, since the energy differences correspond to sub meV/atom, and our dataset with hundreds of frames is not able capture it. This could be a result of using active learning to generate the frames, which would have favored those near the ground-state. To learn the flat energy landscape in more detail, further iterative training starting from each unique stacking is expected to alleviate this issue, but it is not in the scope of the work here to achieve this.

D. Classical transition temperature estimates

In Fig. S38 we show the classical $g(r)$ values for the bilayer, starting from the most stable hollow-filled stacking of the CDWs in the MM stacking. Similarly to the monolayer calculations, clear peaks in the elemental $g(r)$ exists up to 20 K, with higher temperatures only having more subtle deviations from the gaussian fits. It appears that the $g(r)$'s converge to the gaussian fits at lower temperatures than the monolayer case, suggesting a lower transition temperature. Upon visualizing the periodic lattice distortions, we find analogous behavior to the monolayer case, where the hollow phase is relatively stable up to 20 K, while filled phase is only stable below 10 K, with dynamic fluctuations above this temperature.

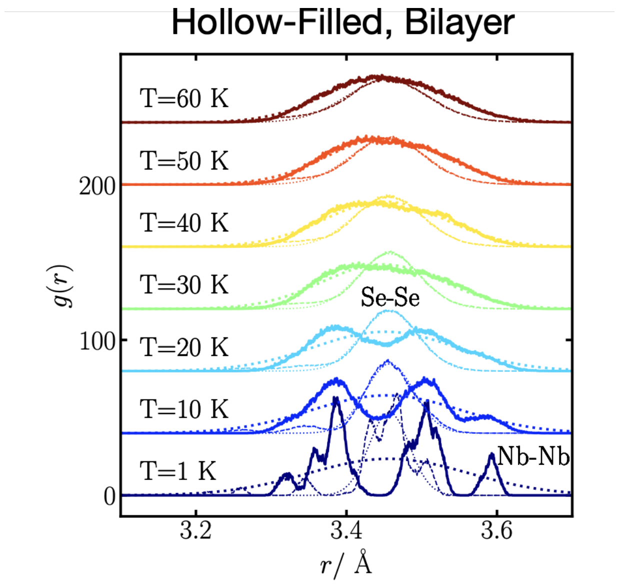

Figure S38: Element resolved $g(r)$ as a function of separation for the indicated temperatures and starting structures.

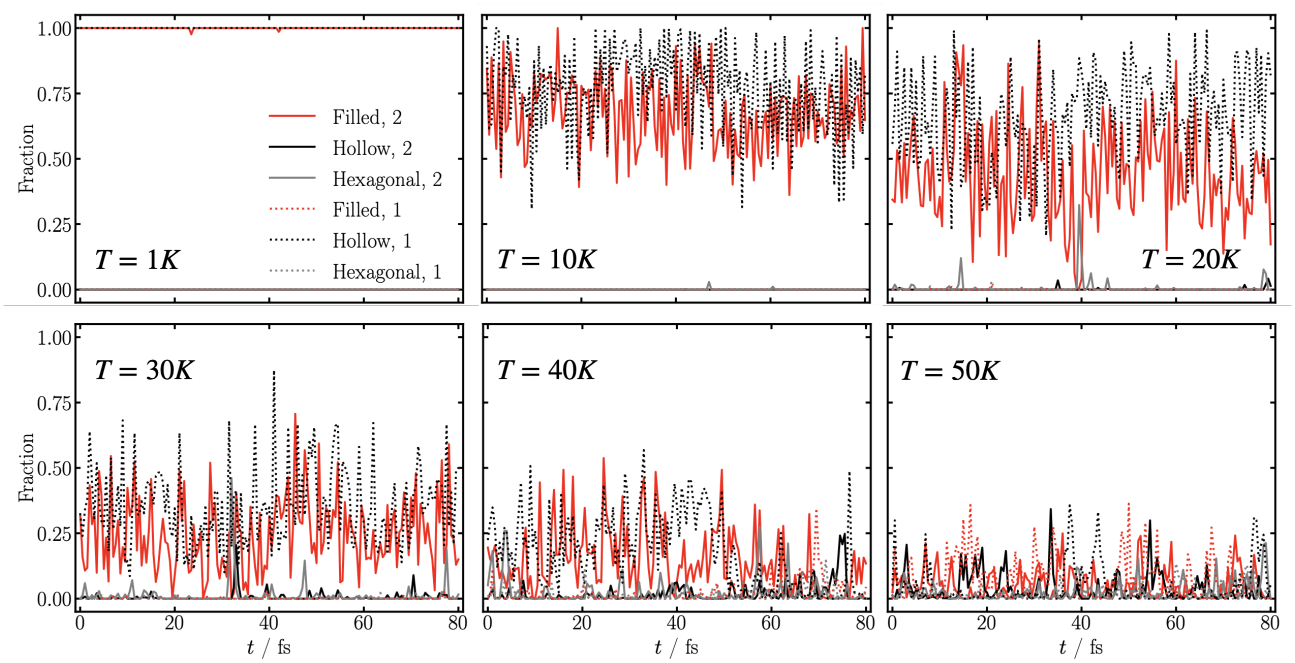

Figure S39: Order parameter from Ref. [23] calculated for each layer as a function of time for the various temperatures.

Again, to understand in more detail the phase behavior as a function of temperature, we computed the order parameter for each phase, following Ref. 23, as seen in Fig. S39. Again, at 1 K, the order parameters of the phases are near 1 for hollow and filled in each layer. At 10 K, the hollow phase has been suppressed slightly more than the monolayer calculation, but the filled phase in the other layer has been stabilized relative to the monolayer. This trend persists to larger temperatures. We also find at the higher temperatures of 50 K, and 60 K (although not shown), that the order parameters of the phases in each layer closer to 0 than in the monolayer, further supporting a lower transition temperature in the bilayer relative to the monolayer.

From the results in Figs. S38 and S39, we place the transition temperature from classical simulations at around 30 K, since this is where slight deviations from gaussian $g(r)$'s are observed, and the order parameters of the phases are consistently less than 1/2.

VII. REFINED MLIP: PHONON DISPERSIONS AND SSCHA CRITICAL TEMPERATURE ESTIMATES

In this section, we further motivate and describe the development MLIPs specifically designed to capture vibrational properties, through tailored datasets and hyperparameters. While the models discussed so far perform well in reproducing energies, forces, and structural reconstructions or CDW dynamics, we have already highlighted their limitations in accurately predicting phonon spectra—and, consequently, related quantities. Below, we expand on these aspects, presenting additional validation tests that guided the refinement of the final models developed for structural predictions, and providing further discussion of the critical temperature estimates beyond classical MD, obtained using the stochastic self-consistent harmonic approximation (SSCHA).

A. Monolayer

As discussed in Section III and in the main text, our best model for the monolayer performs very well for structural properties and related quantities, but still shows limitations in reproducing the vibrational properties of the normal phase. While the agreement between Allegro and DFT is good for a 3×3 supercell—used as a test and proof of concept—it deteriorates for larger ones, needed to fully converge the unstable phonon mode. Notably, the MLIP phonon dispersions are almost insensitive to the supercell size, as shown in Fig. S22(a). This behavior arises from two coupled factors: (i) the current training dataset is dominated by smaller 3×3 supercells, and (ii) the cutoff radius r_{max} spans interactions only within approximately a 3×3 cell. Although transferability to larger supercells is often achieved automatically in standard systems, this is not the case here, as CDW phases involve very small energy differences that are highly sensitive to long-range interactions. Consequently, both the dataset composition and the training strategy (i.e., choice of hyperparameters) must be refined to address this issue.

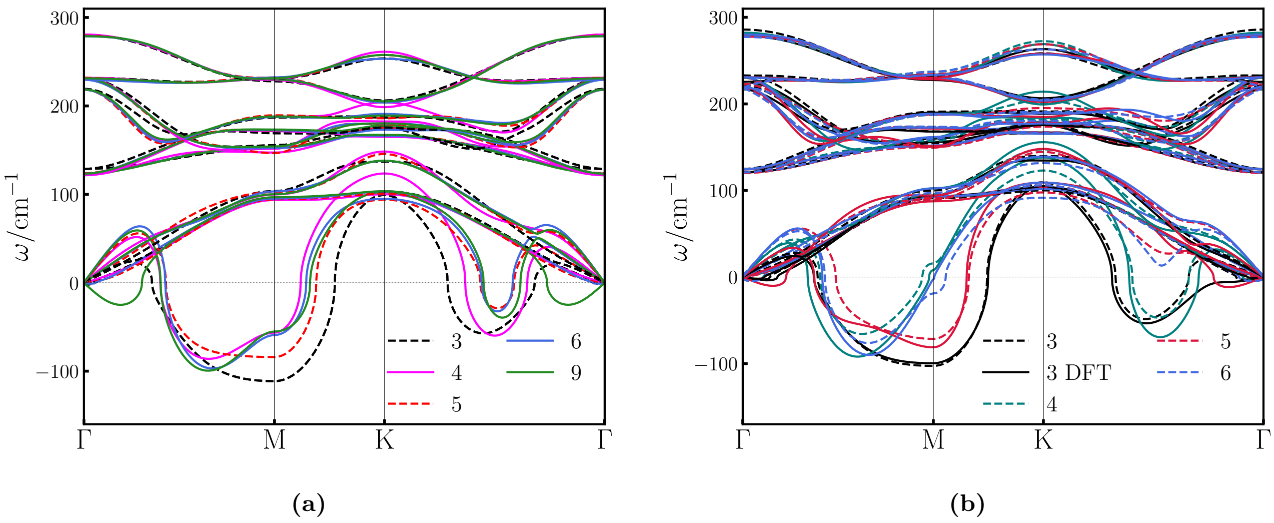

Figure S40: Phonon dispersions of the monolayer after dataset refinements. a, Phonon dispersions of the normal phase obtained with an intermediate MLIP trained on an enlarged dataset including larger supercells and an expanded cutoff radius of 10 Å, covering interactions up to approximately a 6×6 supercell. As discussed in the text, this modified model exhibits an enhanced dependence on supercell size, but residual spurious instabilities remain. **b**, Phonon dispersions obtained with the final refined MLIP, for which the dataset was rebalanced by trimming the smaller (3×3) configurations to achieve a more uniform sampling across supercell sizes. The resulting spectra (dashed lines) show good agreement with DFT (solid lines) up to 6×6 supercells; these are the results discussed in the main text.

As described in the main text, we first increased the number of larger supercells in the dataset while proportionally expanding the cutoff radius to 10 Å, such that interatomic interactions are captured up to roughly a 6×6 supercell—the target size needed to approach a fully converged unstable phonon mode. As shown in Fig. S40(a), this modified MLIP exhibits, as expected, a stronger dependence of the phonon dispersions on the supercell size compared to the prior models. However, these changes were insufficient to achieve satisfactory agreement for the unstable phonon

mode (which can be observed by comparison with DFT results in panel b). The dataset remains biased toward smaller (3×3) configurations, which, as also observed in DFT, display a dominant instability whose minimum is slightly shifted compared to the larger-supercell phonons obtained from DFT+Phonopy or DFPT. The plot clearly shows that spurious instabilities persist even after including additional large supercells and adjusting the cutoff.

To overcome this residual bias, the number of configurations for each supercell size must be rebalanced to achieve a more uniform sampling of supercell sizes. This can be done either by adding more data with larger supercells—although computationally expensive—or by trimming the dataset to reduce the number of smaller supercells; we adopted the latter approach. The results of this refinement, reported in the main text, show that the agreement between DFT and the MLIP is now good up to 6×6 supercells. Beyond this size, further improvements would require extending the dataset to even larger supercells and increasing the cutoff radius accordingly. For comparison, Fig. S40(b) reports the phonon spectra for different supercell sizes obtained with the final MLIP (targeting vibrational properties), whose results are those discussed in the main text.

As discussed in the main text, we used SSCHA [8, 9] to estimate the CDW transition temperature (T_{CDW}). This approach incorporates anharmonic effects and quantum ionic fluctuations—features not captured by the classical MD simulations presented in the previous section.

In Fig. 3 of the main text, we show the temperature-dependent phonon dispersions obtained by combining SSCHA with the refined Allegro model optimized for vibrational properties. The calculations reveal a clear phonon softening near 60 K and the emergence of imaginary (negative) frequencies around 50 K, marking the onset of CDW formation.

To highlight the impact of model quality on temperature-dependent behavior, we also performed SSCHA calculations using an earlier version of the potential (targeting structural properties)—the model employed for phonon comparisons before rebalancing the dataset and extending the real-space cutoff. As shown in Fig. S41(a), this older potential predicts substantially lower transition temperatures: phonon softening appears near 20 K and the instability develops around 10 K, strongly underestimating T_{CDW} relative to both theoretical and experimental benchmarks and remaining close to the classical MD results presented above. This comparison demonstrates how crucial it is to train an MLIP that is not only structurally transferable but also capable of accurately capturing vibrational and anharmonic properties—a key takeaway that, while system-specific, remains broadly relevant for the design and deployment of MLIPs for CDW systems. These results further suggest that the updated potential successfully captures both the correct temperature scale and the momentum dependence of the instability.

For these calculations based on the older model (which is structurally accurate but vibrationally limited), we also analyzed convergence with respect to both the supercell size and the stochastic sampling in SSCHA. These convergence tests were performed on the original potential; however, the same considerations apply to the refined model used for the vibrational analyses discussed in the main text. We examined convergence with respect to the number of configurations N_c used for stochastic sampling in SSCHA. The method refines the anharmonic free-energy surface by averaging over N_c random atomic configurations to compute ensemble averages and gradients. Calculations performed at 10 K—where convergence is most demanding—show that $N_c = 2000$ provides stable and converged results. This value was therefore adopted for all SSCHA simulations.

Finally, Fig. S41(c) compares SSCHA phonon dispersions at 10 K (NVT ensemble) obtained with three earlier potentials (prior to the refinement): (i) the original 552 monolayer model lacking strained and incommensurate structures, (ii) a preliminary 552 variant trained on a limited dataset, and (iii) the bilayer-trained potential (842). Although these models yield similar harmonic phonons at 0 K, differences in the unstable mode lead to markedly distinct finite-temperature behavior. The original monolayer potential fails to reproduce the correct instability wavevector (deviating from 2/3 Γ–M), and the softening vanishes prematurely while a spurious secondary instability emerges along K–Γ. The bilayer-trained potential exhibits comparable shortcomings, overestimating the instability amplitude and misplacing the soft-mode minimum.

In conclusion, these analyses emphasize three main points. (i) Accurate SSCHA predictions require MLIPs explicitly trained to reproduce vibrational properties, not just structural energetics. (ii) The training dataset must include larger, strained, and incommensurate configurations and remain balanced across supercell sizes, with adequate representation of the largest structures. Up to the supercell sizes typically required for convergence (9×9 or 12×12), MLIPs cannot be expected to extrapolate reliably; they must be trained on the specific cell sizes of interest, which may represent a computational bottleneck for certain studies. (iii) Convergence with respect to both supercell size and stochastic sampling is essential for a robust estimation of T_{CDW}. With these refinements, the Allegro-based framework provides a predictive and transferable description of temperature-dependent lattice instabilities in $NbSe_2$.

B. Bilayer

Building on the tests and refinements performed for the monolayer, we applied the same strategy to bilayer $NbSe_2$, as discussed in the main text. There, we highlight that collecting additional configurations for progressively larger su-

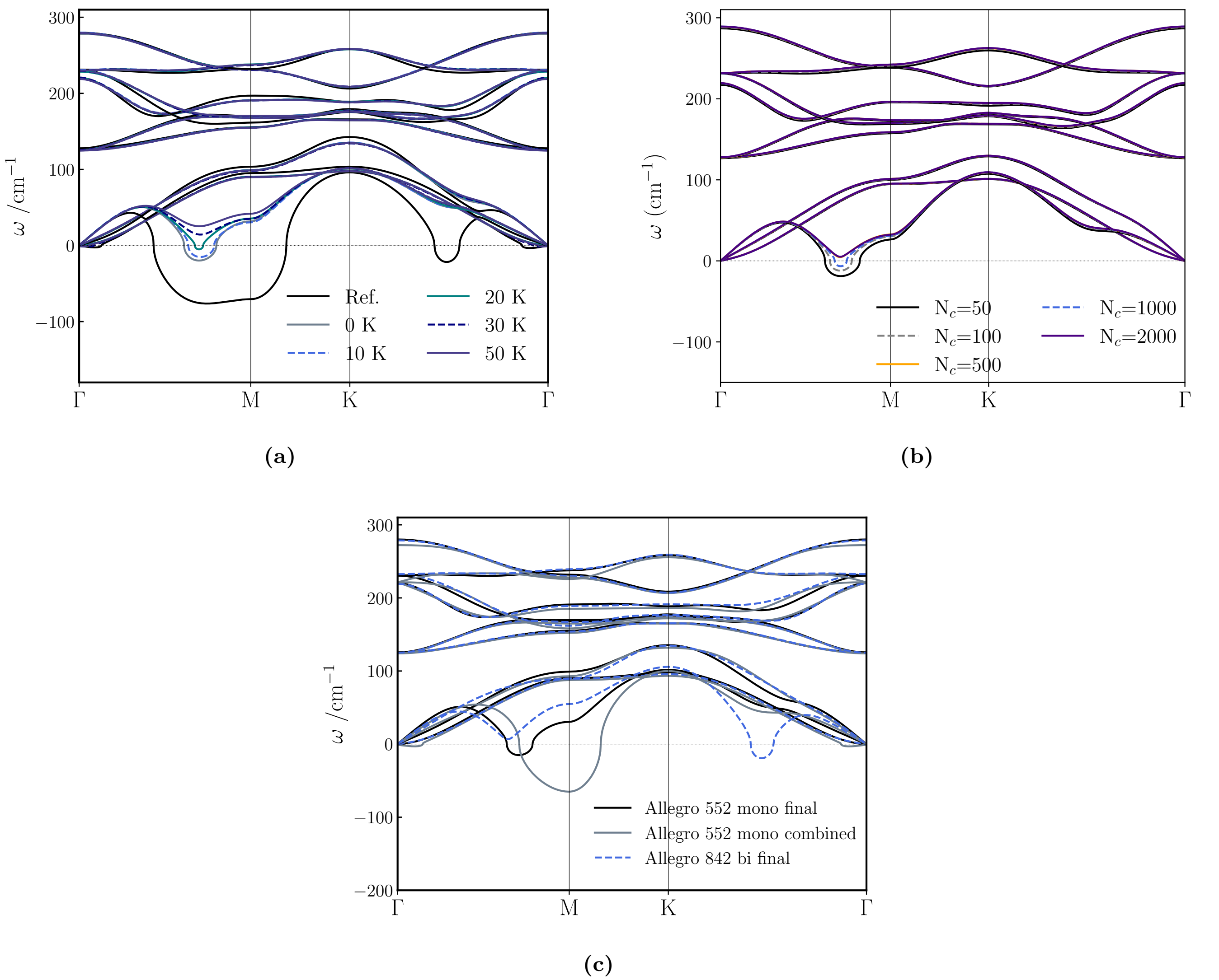

Figure S41: SSCHA phonon dispersions of monolayer $NbSe_2$ obtained with earlier MLIPs. a, Temperature-dependent phonon dispersions calculated using the original (pre-refinement) Allegro model employed for phonon tests before dataset rebalancing and cutoff extension. The softening appears near 20 K and the instability develops around 10 K, strongly underestimating T_{CDW} compared to the refined model discussed in the main text. **b**, Convergence test with respect to the number of stochastic configurations N_c used in the SSCHA sampling at 10 K, showing that $N_c = 2000$ ensures stable and converged results. **c**, Comparison of SSCHA phonon dispersions at 10 K obtained with three earlier MLIPs: (i) the original 552 monolayer model lacking strained and incommensurate configurations, (ii) a preliminary 552 variant trained on a reduced dataset, and (iii) the bilayer-trained potential (842). Differences in the unstable mode and instability wavevector demonstrate how model quality and dataset balance critically affect finite-temperature lattice instabilities.

percell sizes is even more computationally demanding for bilayers and represents a significant bottleneck. Rather than increasing the number of large bilayer supercells, we started from the pruned dataset refined for the monolayer—which satisfactorily reproduces vibrational properties and yields accurate T_{CDW} estimates within SSCHA—and added the bilayer configurations already available. These bilayer frames correspond exclusively to 3×3 supercells.

The rationale was that combining the monolayer data, which carry most of the information about the unstable phonon mode, with a limited number of bilayer configurations, capturing the weak interlayer coupling, could already improve vibrational predictions without extensive new sampling. To this end, we trained several MLIPs on different dataset combinations: (i) the pruned monolayer dataset plus 150 selected bilayer frames (Fig. S42(a)), (ii) the pruned monolayer dataset plus 250 selected bilayer frames (Fig. S42(b)), and (iii) the pruned monolayer dataset plus all the

bilayer frames (Fig. S42(c)), each subset extracted from the existing bilayer dataset.

The results show that it is difficult to find a satisfactory trade-off between accuracy and the number of 3×3 bilayer configurations. Including only a small number of bilayer frames leads to a noticeable deterioration of the overall phonon agreement, while adding enough to recover bilayer features introduces a bias that partially offsets the improvements achieved with the refined monolayer model. Therefore, the best-performing model so far combines the pruned monolayer dataset from the previous section with all bilayer frames currently available. Further improvement would require adding bilayer configurations for larger supercells, up to the 6×6 size needed to fully capture the long-range interactions and reach convergence of the unstable phonon mode.

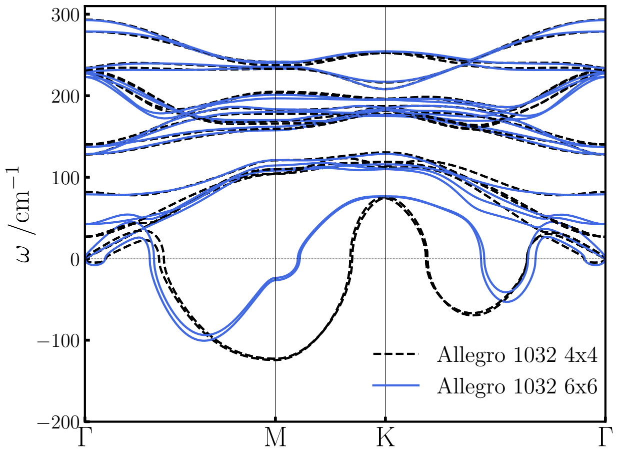

(a)

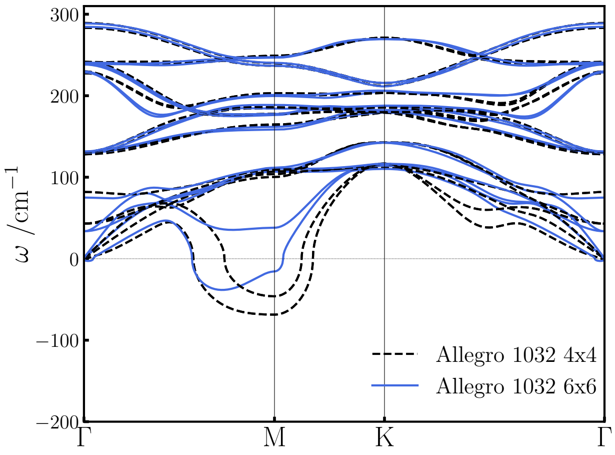

(b)

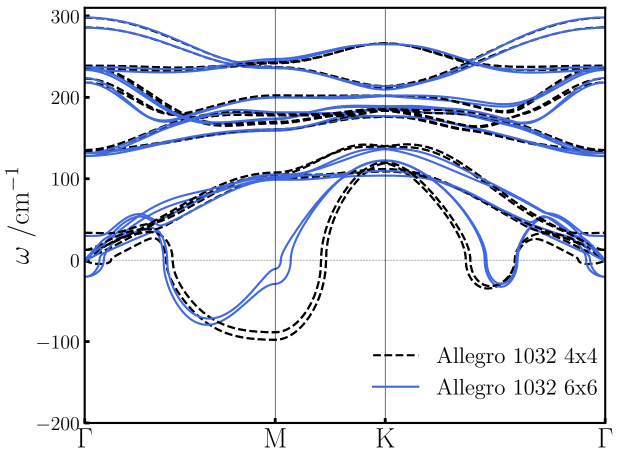

(c)

Figure S42: Phonon dispersions of bilayer $NbSe_2$ obtained with MLIPs trained on combined monolayer and bilayer datasets. a, Phonon dispersions obtained from a model trained on the pruned monolayer dataset combined with 150 selected 3×3 bilayer configurations. **b**, Results for the model trained on the same monolayer dataset plus 250 selected 3×3 bilayer configurations. **c**, Phonon dispersions for the model trained on the pruned monolayer dataset combined with all available 3×3 bilayer frames, corresponding to the best-performing potential discussed in the text. The comparison highlights the difficulty of balancing bilayer accuracy and dataset size: too few bilayer frames lead to degraded overall phonon agreement, while including more frames introduces a bias that partially offsets the improvements achieved with the refined monolayer model.

Following the same approach used for the monolayer, we also present the SSCHA results for the bilayer. The final SSCHA calculations, based on the phonon dispersions and MLIP reported in Fig. S42(c), are presented in the main text. Here, for completeness, we report the results obtained using the older MLIP, prior to the refinements discussed

in this section.

In these simulations, the MLIP was applied to two bilayer stacking configurations: MM, the most stable for antiparallel (180°) alignment and overall lowest-energy structure, and AB, the most stable for parallel (0°) alignment. A 6×6 supercell was used to capture the correct CDW periodicity (i.e., 3×3) and to ensure phonon convergence, consistent with the monolayer calculations.

Similarly to the monolayer, the older potential yields a lower transition temperature for both stackings—around 10 K for the MM configuration (Fig. S43(a)) and slightly lower for the AB stacking (Fig. S43(b))—highlighting the role of stacking in the CDW evolution. Both values remain below the monolayer result, reproducing the weak dimensionality dependence but failing to achieve quantitative agreement with DFT and experimental benchmarks reported in the literature.

Finally, we note that while the refined potential provides a more reliable estimate of the transition temperature within SSCHA, classical MD simulations already yield reasonable results when using the older potentials, albeit within the inherent limitations of this method—namely, the absence of quantum effects, which leads to an underestimation of the transition temperatures in both mono- and bilayers. This is expected, since classical MD captures the transition through structural changes only—effects that were already well described by the earlier models, even without accounting for quantum ionic fluctuations.

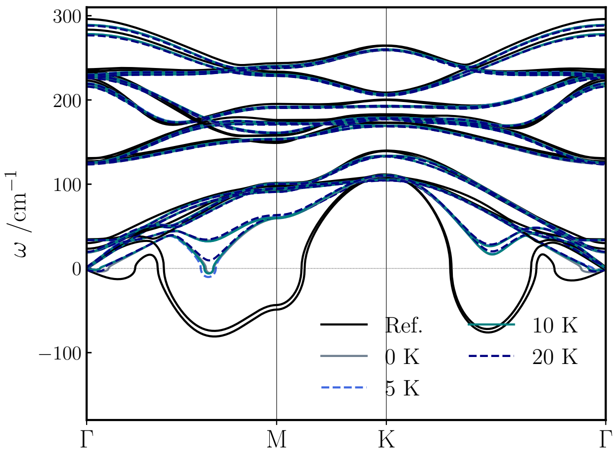

(a)

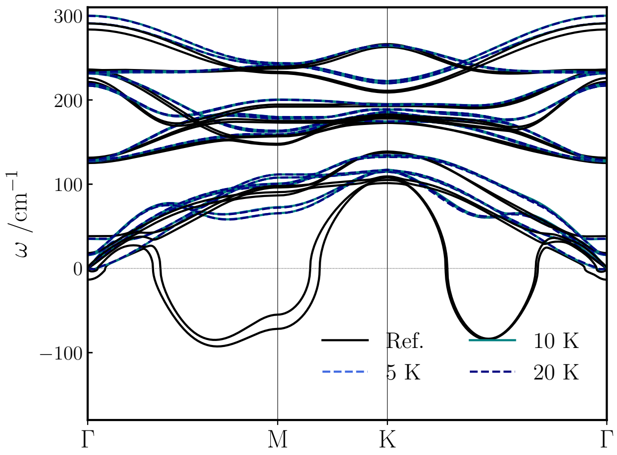

(b)

Figure S43: Temperature-dependent SSCHA phonon dispersions of bilayer $NbSe_2$ obtained with the pre-refinement MLIP. a, Results for the MM stacking, corresponding to antiparallel (180°) alignment, showing a transition temperature of approximately 10 K. **b**, Results for the AB stacking, corresponding to parallel (0°) alignment, yielding a slightly lower transition temperature. Both configurations exhibit values below the monolayer result, reproducing the weak dimensionality dependence of the CDW transition but failing to achieve quantitative agreement with DFT and experimental benchmarks, as discussed in the text.

[1] P. Hohenberg and W. Kohn, Inhomogeneous electron gas, Physical review **136**, B864 (1964).

[2] W. Kohn and L. J. Sham, Self-consistent equations including exchange and correlation effects, Physical review **140**, A1133 (1965).

[3] P. Giannozzi, S. De Gironcoli, P. Pavone, and S. Baroni, Ab initio calculation of phonon dispersions in semiconductors, Phys. Rev. B **43**, 7231 (1991).

[4] A. Musaelian, S. Batzner, A. Johansson, L. Sun, C. J. Owen, M. Kornbluth, and B. Kozinsky, Learning local equivariant representations for large-scale atomistic dynamics, Nat. Commun. **14**, 579 (2023).

[5] A. P. Thompson, H. M. Aktulga, R. Berger, D. S. Bolintineanu, W. M. Brown, P. S. Crozier, P. J. In't Veld, A. Kohlmeyer, S. G. Moore, T. D. Nguyen, *et al.*, Lammps-a flexible simulation tool for particle-based materials modeling at the atomic, meso, and continuum scales, Computer Physics Communications **271**, 108171 (2022).

[6] C. Grindon, S. Harris, T. Evans, K. Novik, P. Coveney, and C. Laughton, Large-scale molecular dynamics simulation of dna: implementation and validation of the amber98 force field in lammps, Philosophical Transactions of the Royal Society of London. Series A: Mathematical, Physical and Engineering Sciences **362**, 1373 (2004).

[7] A. Togo, First-principles phonon calculations with phonopy and phono3py, Journal of the Physical Society of Japan **92**, 012001 (2023).
[8] L. Monacelli, R. Bianco, M. Cherubini, M. Calandra, I. Errea, and F. Mauri, The stochastic self-consistent harmonic approximation: calculating vibrational properties of materials with full quantum and anharmonic effects, J. Phys.: Condens. Matter **33**, 363001 (2021).
[9] R. Bianco, I. Errea, L. Paulatto, M. Calandra, and F. Mauri, Second-order structural phase transitions, free energy curvature, and temperature-dependent anharmonic phonons in the self-consistent harmonic approximation: Theory and stochastic implementation, Phys. Rev. B **96**, 014111 (2017).
[10] P. Giannozzi, S. Baroni, N. Bonini, M. Calandra, R. Car, C. Cavazzoni, D. Ceresoli, G. L. Chiarotti, M. Cococcioni, I. Dabo, *et al.*, Quantum espresso: a modular and open-source software project for quantum simulations of materials, Journal of physics: Condensed matter **21**, 395502 (2009).
[11] P. Giannozzi, O. Andreussi, T. Brumme, O. Bunau, M. B. Nardelli, M. Calandra, R. Car, C. Cavazzoni, D. Ceresoli, M. Cococcioni, *et al.*, Advanced capabilities for materials modelling with quantum espresso, J Phys.: Condens. matter **29**, 465901 (2017).
[12] D. Vanderbilt, Soft self-consistent pseudopotentials in a generalized eigenvalue formalism, Physical review B **41**, 7892 (1990).
[13] V. R. Cooper, Van der waals density functional: An appropriate exchange functional, Physical Review B—Condensed Matter and Materials Physics **81**, 161104 (2010).
[14] M. Methfessel and A. T. Paxton, High-precision sampling for brillouin-zone integration in metals, physical review B **40**, 3616 (1989).
[15] T. Sohier, M. Calandra, and F. Mauri, Density functional perturbation theory for gated two-dimensional heterostructures: Theoretical developments and application to flexural phonons in graphene, Physical Review B **96**, 075448 (2017).
[16] J. Vandermause, S. B. Torrisi, S. Batzner, Y. Xie, L. Sun, A. M. Kolpak, and B. Kozinsky, On-the-fly active learning of interpretable bayesian force fields for atomistic rare events, npj Comput. Mater. **6**, 20 (2020).
[17] R. Drautz, Atomic cluster expansion for accurate and transferable interatomic potentials, Phys. Rev. B **99**, 014104 (2019).
[18] C. Lin, S. Poncé, and N. Marzari, General invariance and equilibrium conditions for lattice dynamics in 1d, 2d, and 3d materials, npj Computational Materials **8**, 236 (2022).
[19] X. Gonze and C. Lee, Dynamical matrices, born effective charges, dielectric permittivity tensors, and interatomic force constants from density-functional perturbation theory, Phys. Rev. B **55**, 10355 (1997).
[20] S. Baroni, S. De Gironcoli, A. Dal Corso, and P. Giannozzi, Phonons and related crystal properties from density-functional perturbation theory, Rev. Mod. Phys. **73**, 515 (2001).
[21] A. Stukowski, Visualization and analysis of atomistic simulation data with ovito–the open visualization tool, Model. Simul. Mater. Sci. Eng **18**, 015012 (2009).
[22] R. Bianco, L. Monacelli, M. Calandra, F. Mauri, and I. Errea, Weak dimensionality dependence and dominant role of ionic fluctuations in the charge-density-wave transition of $NbSe_2$, Phys. Rev. Lett. **125**, 106101 (2020).
[23] C. T. S. Cheung, Z. A. H. Goodwin, Y. Han, J. Lu, and A. A. M. andJohannes Lischner, Coexisting charge density waves in twisted bilayer $NbSe_2$, Nano Lett. **24**, 12088 (2024).
